# Supplementary material for: Pharmacologically reversible zonation-dependent endothelial cell transcriptomic changes with neurodegenerative disease associations in the aged brain
Source: Nat Commun. 2020 Sep 4;11:4413. doi: 10.1038/s41467-020-18249-3 (PMC7474063; doi:10.1038/s41467-020-18249-3)
Supplement: Supplementary file 1 — Supplementary Information [file 41467_2020_18249_MOESM1_ESM.pdf]

Supplementary Information

Pharmacologically reversible zonation-dependent endothelial cell transcriptomic changes with neurodegenerative disease associations in the aged brain

Zhao et al.

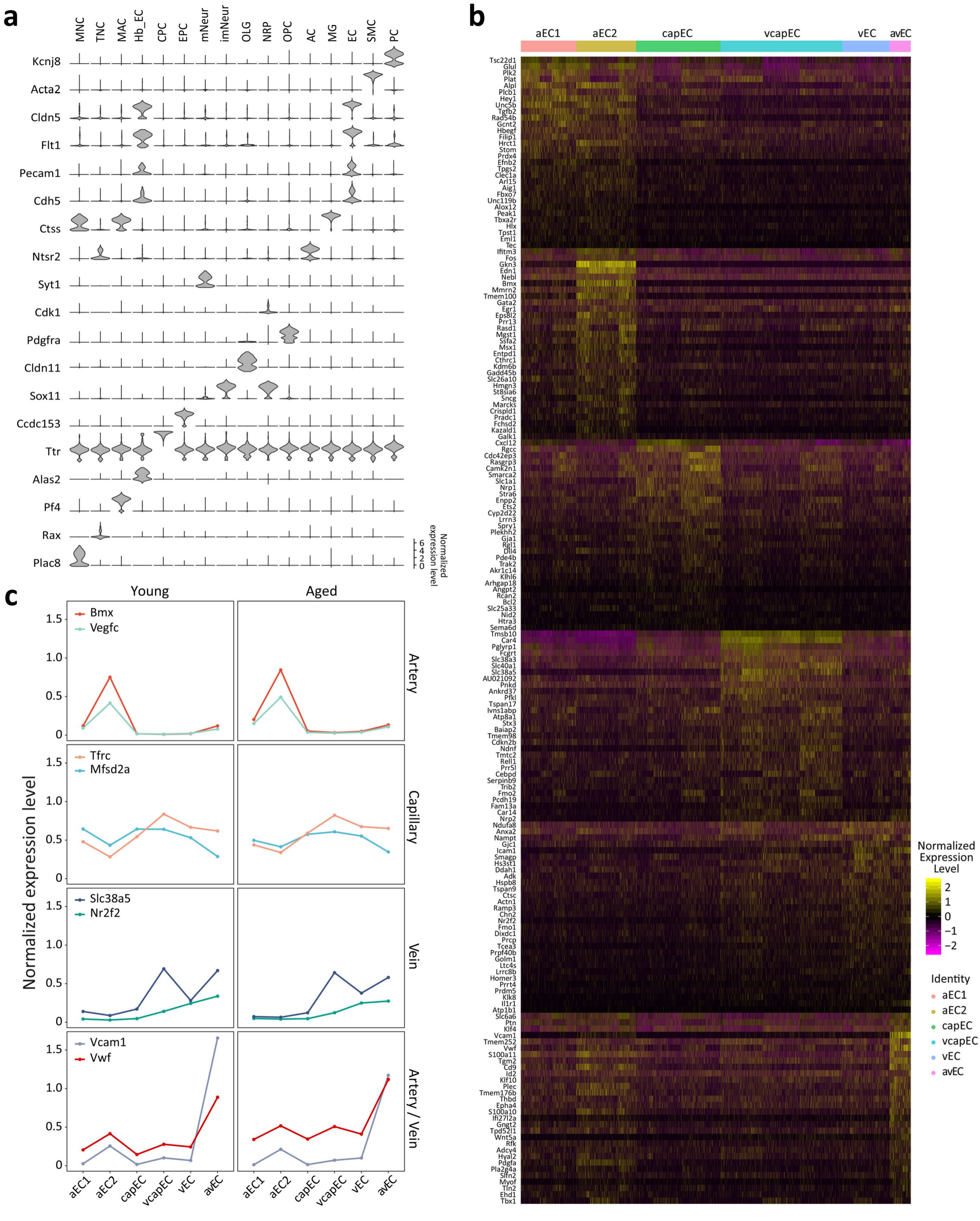

Supplementary Fig. 1. Marker gene expression patterns in different cell types and subtypes. a, Violin plots of the expression patterns of marker genes for major cell type clusters identified. Each column corresponds to one primary cell type. Cell type abbreviations: same as **Fig. 1b**. **b,** Heatmap showing the expression patterns of variable genes along the arteriovenous axis employed for classification of EC subtypes (see Methods). Up to 30 genes are shown for each EC subtype. **c,** Relative average expression levels of marker genes of artery/arteriole (*Bmx* and *Vegfc*), capillary (*Tfrc* and *Mfsd2a*), vein (*Slc38a5* and *Nr2f2*), and artery/vein (*Vcam1* and *Vwf*) in the EC subtypes.

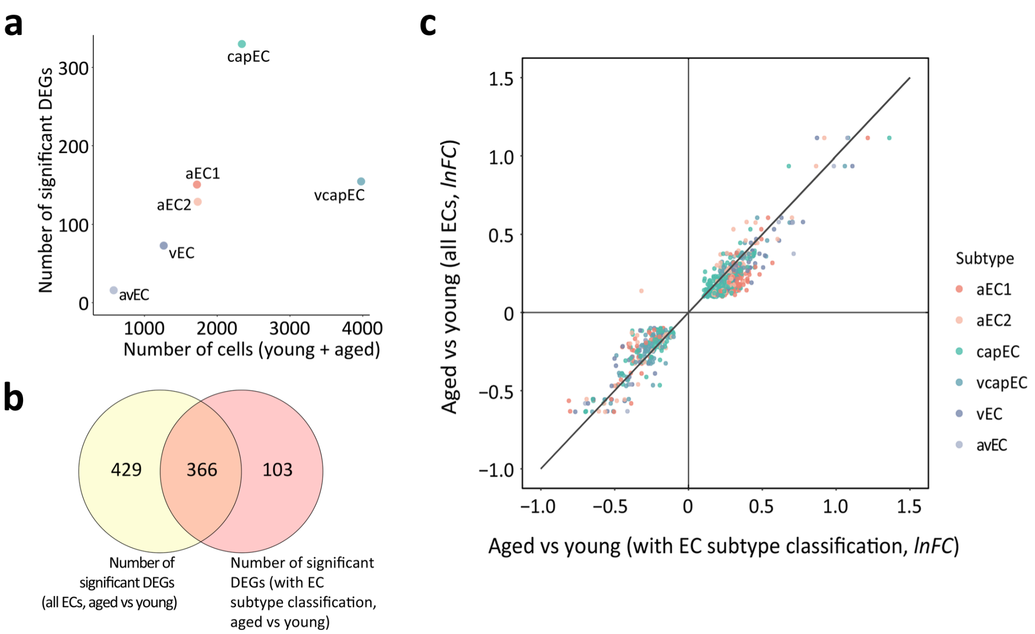

Supplementary Fig. 2. Dependence of differential expression analysis on EC subtype classification and cell number. a, Number of significant DEGs plotted against total number of cells sampled (young adult + aged groups) for each EC subtype. **b,** Venn diagram of EC-subtype DEGs and pooled-EC DEGs. The expression changes of a substantial proportion (22.0%, 103 out of 469) of DEGs would not be detected without subtype classification. **c,** *lnFC* in EC subtypes (x-axis) plotted against *lnFC* in all ECs pooled (y-axis) for DEGs significant in both subtype- and pooled EC-based comparisons across age groups. Note that genes with significant differential expression in more than one EC subtype have multiple points. For up- or downregulated DEGs, the majority of points (60.1%, 424 out of 706, $P = 4.5 \times 10^{-8}$ compared to chance level by one-sided, one proportion z-test) fall below or above the line with unit slope respectively.

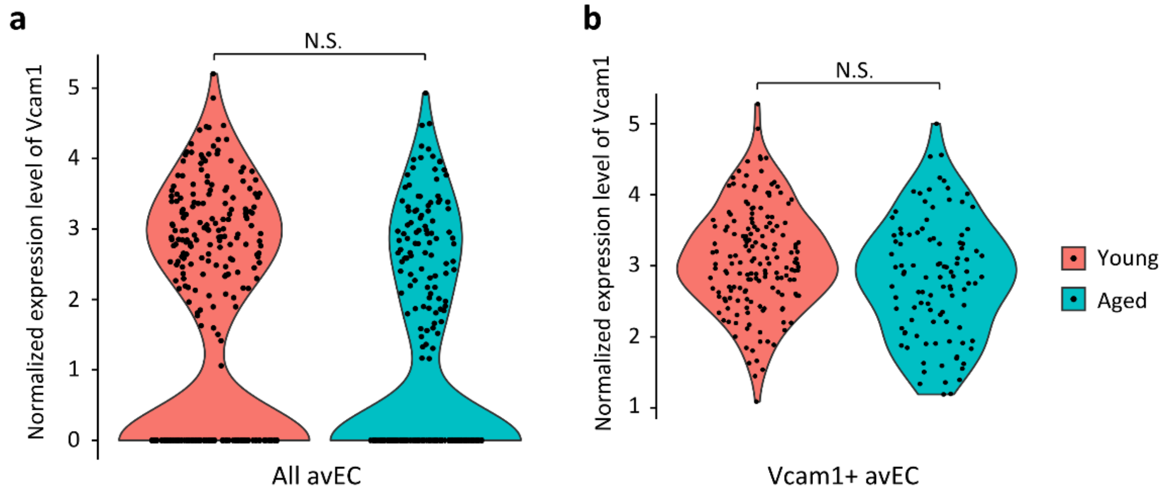

Supplementary Fig. 3. *Vcam1* expression level in the avEC subtype across age. Violin plots of *Vcam1* expression levels compared across age for **a,** all avECs (*lnFC* = -0.39; FDR-adjusted *P*-value = 1), or **b,** avECs with non-zero *Vcam1* transcript count (*lnFC* = -0.19; FDR-adjusted *P*-value = 1).

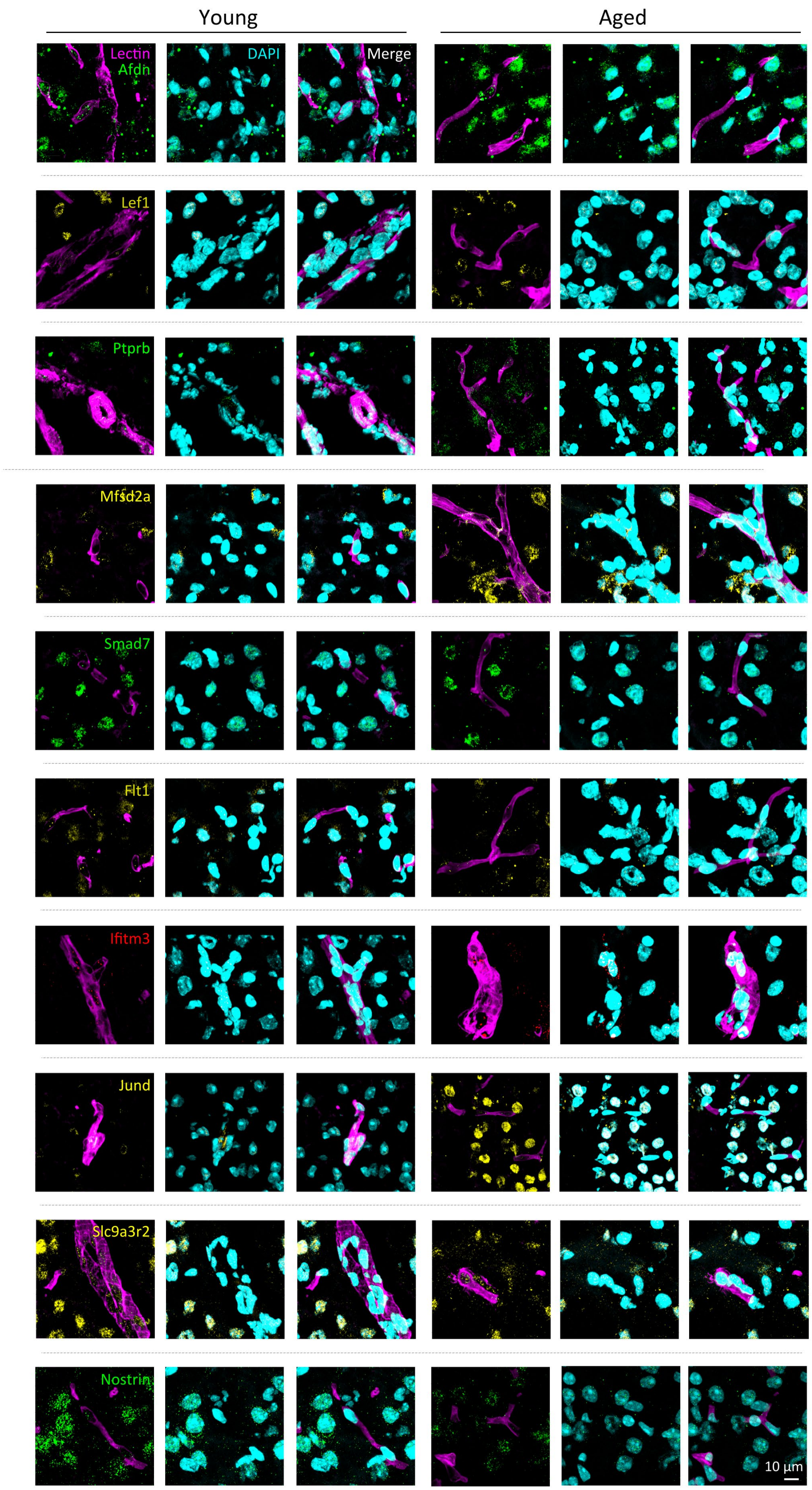

Supplementary Fig. 4. RNA fluorescent *in situ* hybridization (FISH) of selected genes. RNA FISH of selected genes (one gene per row, from top to down: *Afdn*, *Lef1*, *Ptprb*, *Mfsd2a*, *Smad7*, *Flt1*, *Ifitm3*, *Jund*, *Slc9a3r2*, *Nostrin*) in young adult (left three columns) and aged (right three columns) mouse brain sections, co-stained with lectin and DAPI for visualization of endothelium and cell nuclei (number of staining experiments carried out for each target gene: 2).

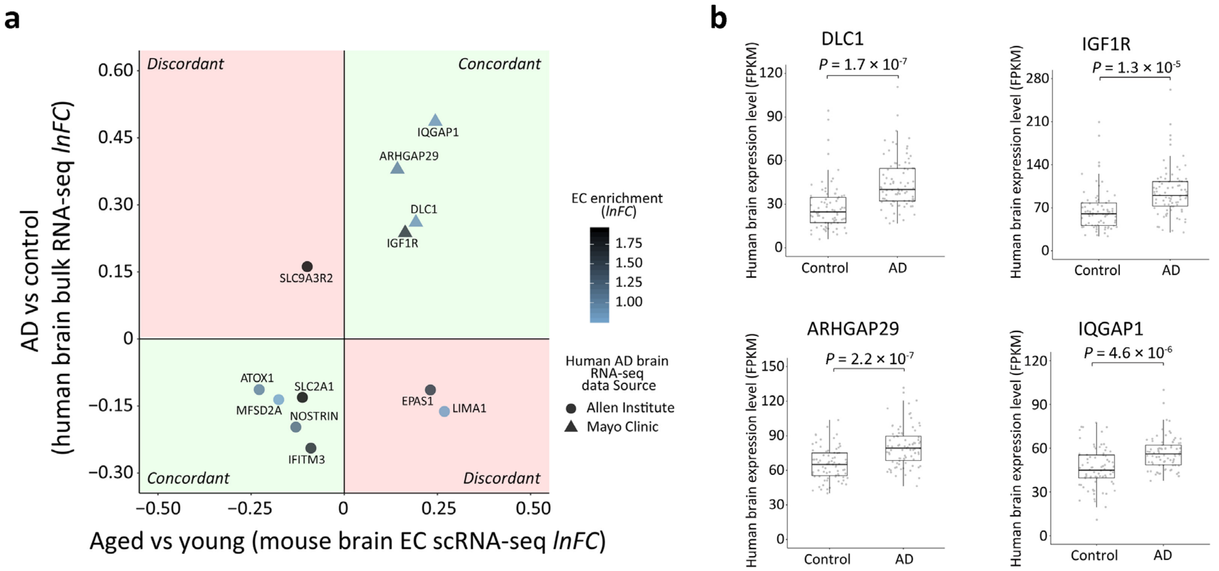

Supplementary Fig. 5. Supplementary comparative analysis with the Mayo Clinic AD brain bulk RNA-seq dataset. a, Similar to **Fig. 3b** but with additional genes from comparative analysis using the Mayo Clinic AD brain bulk RNA-seq dataset. Human AD brain differential expressions relative to age-matched control subjects (FDR-adjusted *P*-value < 0.05, y-axis) plotted against expression changes in pooled aged mouse brain ECs (x-axis), showing aged mouse brain EC DEGs whose human orthologs had concordant (first and third quadrants, light green) or discordant (second and fourth quadrants, light red) expression changes in human AD brains. Only DEGs with at least two-fold EC-enrichment (*lnFC* of EC expression relative to other cell types > 0.7) are shown, with color of dots representing the degree of enrichment. **b,** Human brain expression levels of genes with concordant expression changes in human AD and aged mouse brains identified from the Mayo Clinic AD brain bulk RNA-seq dataset ($n = 78$ samples from control brains, 82 samples from AD brains; two-sided unpaired *t*-test with adjustment for multiple comparisons). Black horizontal line: median; upper and lower bounds of box: 75th and 25th percentiles respectively; upper and lower bounds of vertical lines: upper quartile + 1.5 × interquartile range or maximum (whichever is smaller), and lower quartile - 1.5 × interquartile range or minimum (whichever is larger), respectively.

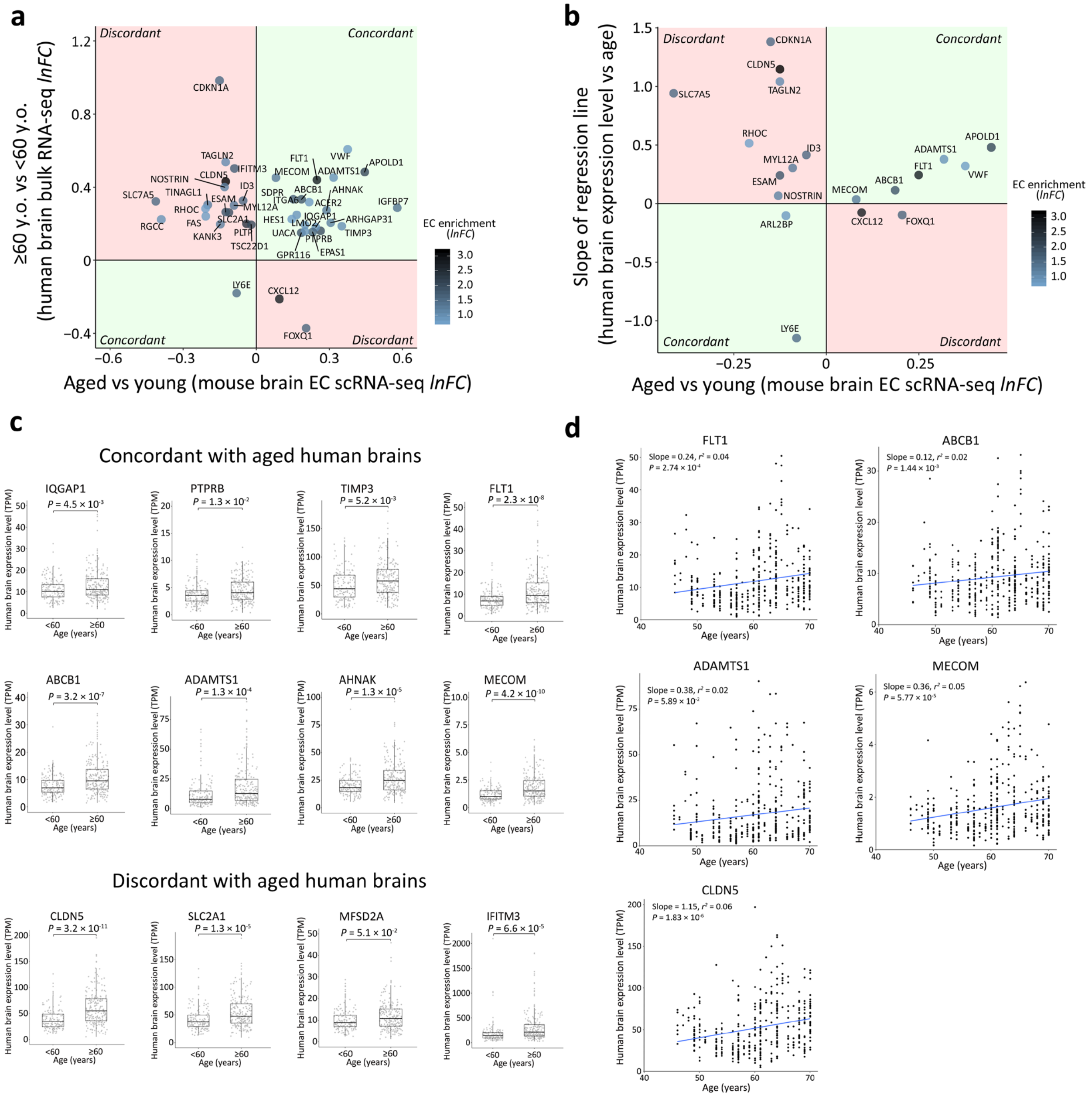

Supplementary Fig. 6. Comparative analysis with normal human aging brain data from the Genotype-Tissue Expression (GTEx) project database. a, Aged human brain (≥ 60 years old) differential expressions relative to younger subjects (< 60 years old) (FDR-adjusted P-value < 0.05, y-axis) plotted against expression changes in pooled aged mouse brain ECs (x-axis), showing aged mouse brain EC subtype DEGs whose human orthologs had concordant (first and third quadrants, light green) or discordant (second and fourth quadrants, light red) expression changes in aged human brains (GTEx dataset v7). Only DEGs with at least two-fold enrichment in EC (i.e. *lnFC* of expression in ECs relative to other cell types > 0.7) were included, with color of dots representing the degree of EC enrichment. **b,** Similar to **a.**, but with y-axis being the slope of regression of expression level with respect to age (GTEx dataset v8). Only genes with significant slope on linear regression (i.e. $P < 0.05$) are included. **c,** Human brain expression levels of selected genes associated with neurovascular regulation (*Iqgap1*, *Ptprb*, *Timp3*, *Flt1*) and AD (P-gp gene, *Adamts1*, *Ahnak*, *Mecom*) with concordant changes in aged human and mouse brains (≥ 60 versus < 60 years old human brains, FDR-adjusted P-value < 0.05), or with important functional roles at the BBB and yet discordant changes (≥ 60 versus < 60 years old human brains, *Cldn5*, *Slc2a1* and *Ifitm3*: FDR-adjusted P-value < 0.05; *Mfsd2a*: FDR-adjusted P-value = 0.0505, n = 215 samples from 38 ≥ 60 years old brains, 147 samples from 23 < 60 years old brains, GTEx dataset v7; two-sided unpaired t-test with FDR-adjustment for multiple comparisons). Black horizontal line: median; upper and lower bounds of box: 75th and 25th percentiles respectively; upper and lower bounds of vertical lines: upper quartile + 1.5 × interquartile range or maximum (whichever is smaller), and lower quartile - 1.5 × interquartile range or minimum (whichever is larger), respectively. **d,** Human brain expression levels of selected genes plotted against age and the respective linear regression fits (n = 388 samples from 61 subjects, GTEx dataset v8, P-values for linear regression shown are for each gene independently without adjustment for multiple comparisons).

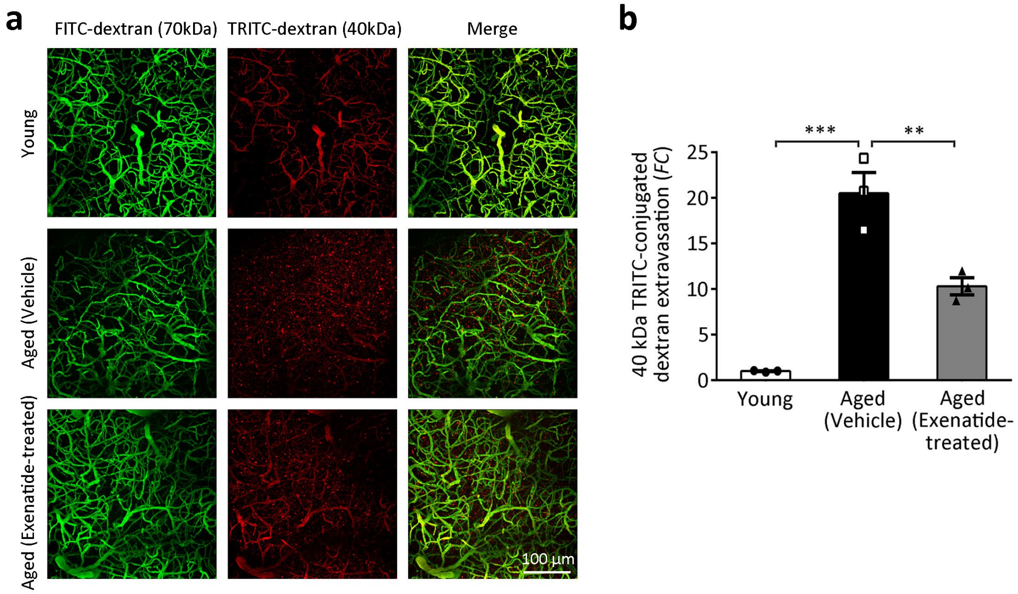

Supplementary Fig. 7. Confirmation of the blood-brain barrier (BBB) rescue effect of exenatide over saline vehicle. a, Three-dimensional rendered images (top view) of *in vivo* two-photon imaging of cerebral vasculature and blood-brain barrier (BBB) leakage in the mouse somatosensory cortex by co-injection of 70 kDa FITC-conjugated dextran (FITC-dextran, green) and 40 kDa TRITC-conjugated dextran (TRITC-dextran, red). FITC-dextran remained in the vasculature and allowed reconstruction of vessels, while extravasation of TRITC-dextran served as an indicator of BBB leakage which was quantified for young adult, saline vehicle- and exenatide-treated aged mouse groups. **b,** Volumetric quantification of TRITC-dextran extravasation showing BBB breakdown in saline vehicle-treated aged (18 – 20 months old) relative to young adult mice (2 – 3 months old) (mean fold change (*FC*) in volume of extravasated TRITC-dextran relative to young adult group ± S.E.M. = 20.5 ± 2.3; $P = 1.7 \times 10^{-4}$ for saline vehicle-treated aged vs young adult mouse group, 3 image stacks were acquired to obtain the mean for each animal, $n = 3$ mice for each group, one-way ANOVA with Tukey's post-hoc test), which was significantly reduced by exenatide treatment (5 nmol/kg/day I.P. for 4 – 5 weeks starting at 17 – 18 months old, mean fold change relative to young adult group ± S.E.M. = 10.3 ± 0.9; $P = 5.4 \times 10^{-3}$ for exenatide-treated vs saline vehicle-treated aged mouse group, 3 image stacks were acquired to obtain the mean for each animal, $n = 3$ mice for each group, one-way ANOVA with Tukey's post-hoc test). Source data are provided as a Source Data file.

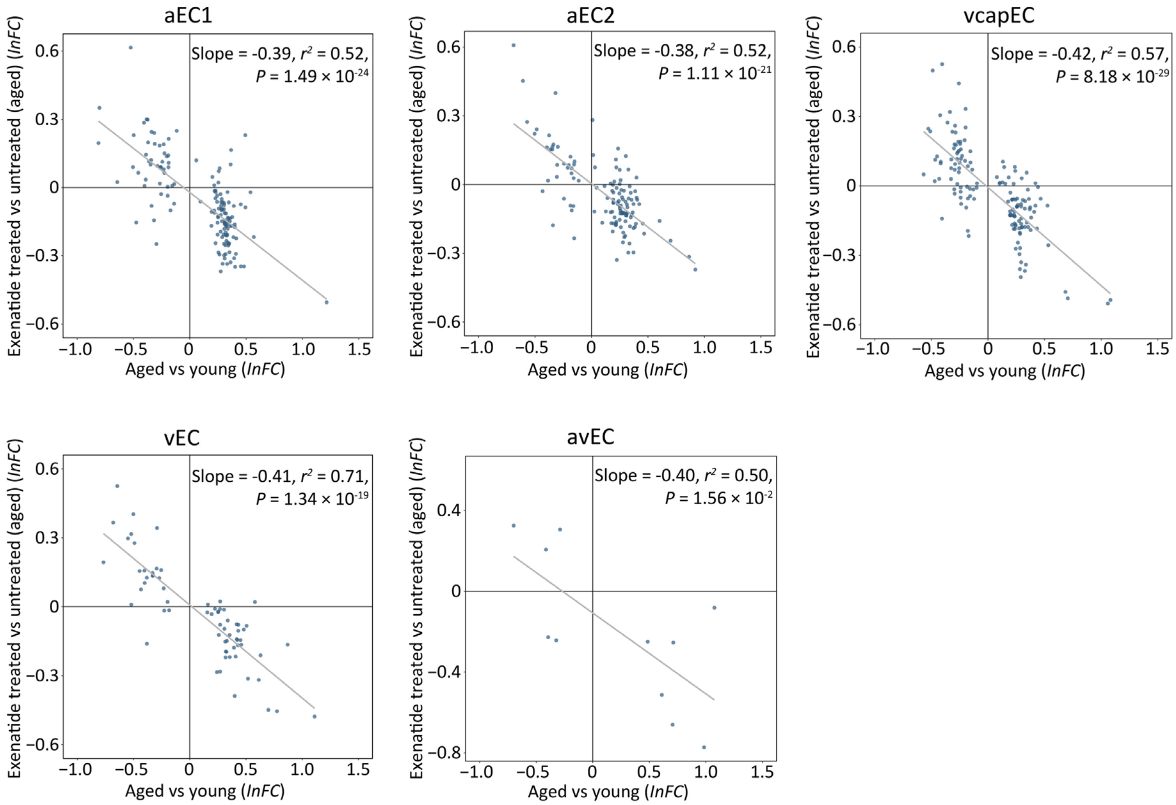

Supplementary Fig. 8. Reversal of ageing-associated transcriptomic changes by exenatide treatment in the EC subtypes. *P*-values of linear regression shown are for each cell subtype independently without adjustment for multiple comparisons.

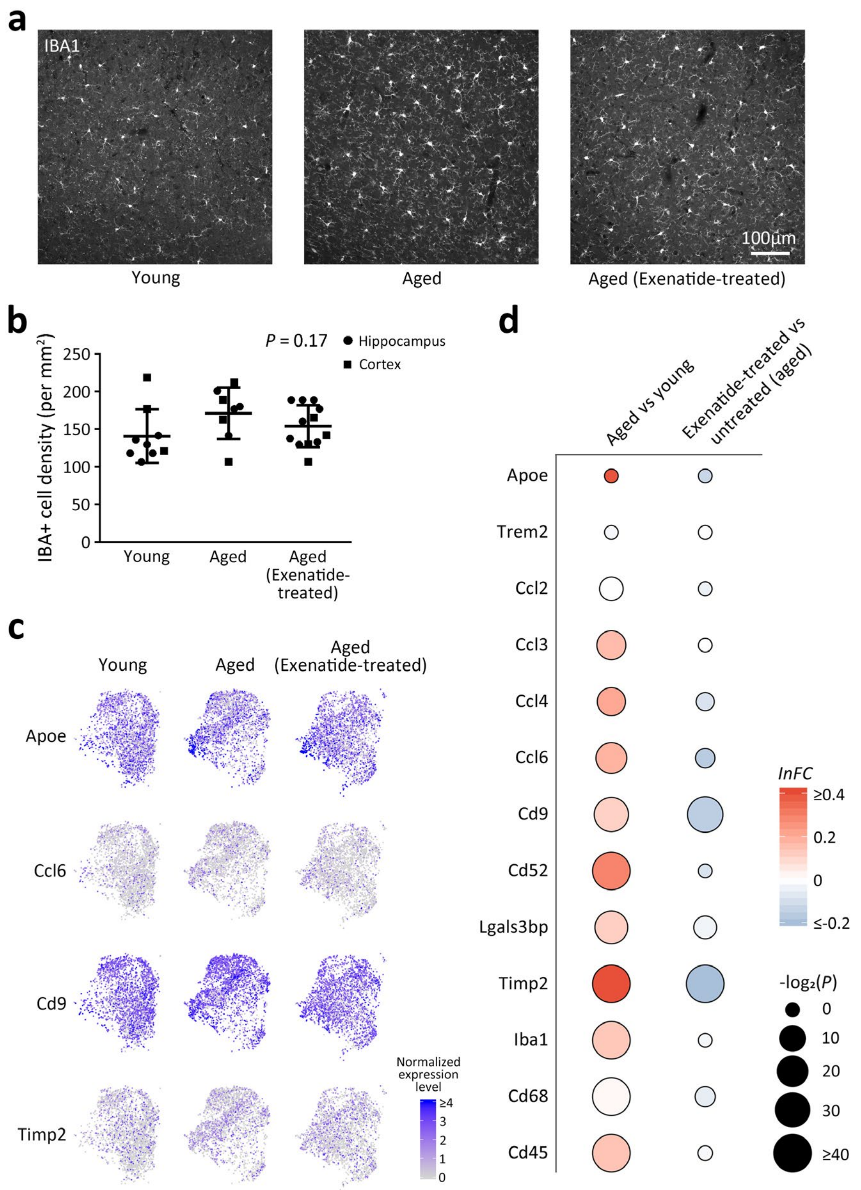

Supplementary Fig. 9. Attenuation of microglial ageing- and neurodegenerative disease-associated signatures by exenatide treatment. a, IBA1 immunostaining in young adult, aged, and exenatide-treated aged mouse cortex. **b,** IBA1+ cell density across the different groups, revealing a trend of increased IBA1+ microglia density in the aged brain which was reduced by exenatide treatment (mean IBA1+ cell density ± S.D. = 141 ± 36 per mm^2 in young adult group, 171 ± 34 per mm^2 in aged group, 154 ± 28 per mm^2 in exenatide-treated aged group brain sections; *n* = 9, 8 and 12 imaged hippocampal and cortical regions of 4, 3 and 3 mice from young adult, aged and exenatide-treated aged groups respectively; $P = 0.17$, one-way ANOVA). Source data are provided as a Source Data file. **c,** t-SNE visualization of the expression of four ageing- and neurodegenerative disease-enriched genes in microglia (*Apoe*, *Ccl6*, *Cd9*, *Timp2*) in the different groups. **d,** Dot plots showing the differential expressions of a panel of microglial ageing-, neurodegenerative disease- and activation-associated genes in the aged mouse brain (relative to young adult group), and the effects of exenatide treatment.

Supplementary Table 1. Arteriovenous zonation marker genes used for EC subtype classification.

EC subtype	Marker genes (source: Nature. 2018 Feb 22;554(7693):475-480[1].)
aEC1	*Sncaip/Alpl/Alox12/B4galnt1/Glul/Syt15/Tpgs2/Fam198b/Antxr1/Eml1/Gm609/Rnf144a/Stom/Prdx4/Aig1/Arhgef25/Stc1/Plat/Peak1/Tec/Efnb2/Fbxo7/Unc5b/Tpst1/Hey1/Cpm/Hrct1/Unc119b/Hbegf/Tbxa2r/Spry4/Scube2/Plcb1/Hlx/Arl15/Tsc22d1/Filip1/A430090L17Rik/Plk2/Slc36a1/Sez6/Rad54b/Gcnt2/Clec1a/Tgfb2/Slc12a5*
aEC2	*Egr1/Mgst1/St8sia2/Entpd1/Zbp1/Cables2/Cthrc1/Msx1/St8sia6/Cbr3/Irf6/Lcat/Olfml2a/Slc27a3/Col18a1/Fchsd2/Prkcd/Ifitm3/Mmrn2/Gata2/Fos/Marcks/Edn1/Kdm6b/Eps8l2/P2ry2/Pfkfb3/Snx10/Ssfa2/Crispld1/Prr13/Atl2/Galk1/Kazald1/Dsp/Sncg/Rasd1/Nebl/Gadd45b/Lama3/Bmx/Gkn3/Tmem100/Ssbp2/Hmgn3/Slc26a10/Pradc1*
capEC	*Dll4/Rasgrp3/Akr1c14/Slc25a33/Smarca2/Ets2/Rcan2/Sema6d/Gja1/Enpp2/Angpt2/Col4a3/Prdm1/Cdc42ep3/Hdac9/Pde4b/Arhgap18/Bcl2/Htra3/Gpr85/Cyp2d22/Itga4/Trak2/Nrp1/Camk2n1/Nid2/Lrrn3/Plekhh2/Rgcc/Stra6/Rgl1/Pcx/Slc1a1/Osgin1/Klhl6/Cxcl12/Spry1*
vcapEC	*Nrp2/Frmd5/Tmem98/Pnkd/Atp8a1/Tmem37/Stx3/Pak1/Pitpnm2/Car14/Jak3/Tspan17/Serpinb9/Unc13c/Fcgrt/Pglyrp1/Kcp/Slc40a1/Slc38a3/Ctla2b/Tmtc2/Irf5/Nos2/Rell1/Hcn2/Coro2b/Baiap2/Rab4a/Pfkl/Trib2/Prr5l/Tmsb10/Ndnf/Car4/Slc38a5/Fmo2/Cebpd/St6gal1/AU021092/Pcdh19/Igsf5/Odc1/Fam13a/Tbx4/Ankrd37/Pmaip1/Grb7/Ivns1abp/Cdkn2b*
vEC	*Dixdc1/Nr2f2/Lbp/Adk/Ltc4s/Tspan9/Tcea3/Ndufa8/Lrrc8b/Chn2/Prcp/Icam1/Il1r1/Tbc1d2b/Nckap5/Gm7694/Bst1/Sdk1/Lcn2/Fmo1/Ctsc/Gpr182/Gm5127/Cysltr1/Hspb8/Prpf40b/Rp1/Mxra8/Prdm5/Itga3/Hs3st1/Anxa2/Golm1/Homer3/Atp1b1/Nampt/Prrt4/Sytl2/Gjc1/Actn1/Klk8/Ddah1/Smagp/Ramp3*
avEC	*Tmem176b/Tgtp1/Id2/Ptn/Gngt2/Tpd52l1/Frem2/Hyal2/Ifi27l2a/Tln2/Slc6a6/Car7/Vcam1/Klf4/Rfk/P2ry1/S100a10/Ehd1/Plec/S100a11/Pla2g4a/Cd9/Pdgfa/Ntn1/Thbd/C530008M17Rik/Aldh1a3/Kcnb1/Tnfrsf11a/Klf10/Carhsp1/Aldh1a1/Wnt5a/Icosl/Cfb/Myof/Adh1/Epha4/Tgm2/Slfn2/Vwf/Adcy4/Tmem252/Tbx1*

Supplementary Table 2. GWAS genes used for disease association analysis.

Disease / condition	GWAS genes	Source(s) of GWAS genes
White matter hyperintensity burden / cerebral small vessel disease	*TRIM65/EFEMP1/TRIM47/PLEKHG1/PDCD11/NEURL1/SH3PXD2A/PMF1/NBEAL1/NRCAM/DAB1/PTPRD/EVL/ZNF16/CHRM3/RASSF3/COL4A2/AHCYL2/C10orf143/PCDH7/QRICH2/KCNB1/DPPA3/ARID1A/VCAN/ITPRID1/PMF1-BGLAP/C1QL1/GAS1*	GWAS Catalog[2]; Circ Cardiovasc Genet. 2015 Apr;8(2):398-409[3].
Stroke	*UBE2E3/FOXF2/OPRM1/MPDZ/CFL2/SYNE2/ALCAM/CDH6/CASC10/NINJ2/SPRY2/F5/PITX2/F2/LRAT/SH2B3/HDAC9/FUNDC2/ALDH2/PMF1BGLAP/ABCC1/ZFHX3/FGB/LRCH1/PATJ/CHD3/SLC44A2/CASZ1/PROCR/FAF1/CDK6/SLCO1B1/ILF3/CDKN2C/PRPF8/PDE3A/ADAMTS13/SMARCA4/DAB1/AGBL1/KIF26B/COX7A2L/SWAP70/CDKN1A/PTPRD/TSPAN15/ALDH1A2/IRX1/SLC26A11/PTPRG/KCNK1/HPS4/MYRIP/C10orf143/PCDH7/QRICH2/CDC5L/CRISP2/KCNK3/DPPA3/ARID1A/NRCAM/LAMA1/WNT2B/TTBK1/RPH3A/ONECUT2/LMNA/RAB19/VWF/FUT8/ADGRF1/KAT2B/FGG/PITX2/ATXN2/TWIST1/PMF1/FGA/ABO/SSPN/TCF7L2/ANK2/FURIN/SH3PXD2A/RHAG/CDH13/ATP6V1G3/KNG1/SLC22A7/APOD/C17orf102/USP38/TNFRSF21/RASEF*	GWAS Catalog[2]; Lancet Neurol. 2016 Jun;15(7):695-707[4].
Parkinson's disease	*GBA/NUCKS1/SLC41A1/ITPKB/SIPA1L2/IL1R2/TMEM163/CCNT2/SCN3A/STK39/SATB1/NCKIPSD/CCDC71/ALAS1/TLR9/DNAH1/BAP1/PHF7/NISCH/STAB1/ITIH3/ITIH4/MCCC1/TMEM175/DGKQ/FAM200B/CD38/FAM47E/SNCA/ANK2/CAMK2D/ELOVL7/ZNF184/HLAQA1/KLHL7/NUPL2/GPNMB/CTSB/MICU3/SORBS3/PDLIM2/C8orf58/BIN3/SH3GL2/FAM171A1/BAG3/DLG2/LRRK2/OGFOD2/GCH1/TMEM229B/GALC/VPS13C/COQ7/ZNF646/KAT8/TOX3/ATP6V0A1/PSMC3IP/TUBG2/ARHGAP27/CRHR1/SPPL2C/MAPT/STH/KANSL1/SYT4/LSM7/DDRGK1/CHMP2B/KRTCAP2/CNTN1/RAB29/CCDC62/BST1/INPP5F/WNT3/HLADRB1/RIT2/GAK/NDUFAF2/NSF/DCUN1D1/HLADQB1/BCKDK/LAMTOR2/SLC2A13/LHFPL2/TIAL1/LZTS3/MAP4K4/MMRN1/TPM1/GPR65/SYT17/HLADRA/IP6K2/PLPPR1/ITGA8/TBC1D5/PMVK/SCN2A/ITIH1/KLHDC1/SLC50A1/COL3A1/CA8/PLEKHM1/CCN6/SREBF1/TCEANC2/AGAP1/CYP17A1/CCDC82/TMC3/PAM/ZNF165/GBF1/KTN1/MX2/CAB39L/TAS1R2/QSER1/COL13A1/TMPRSS9/ZP3/CNKSR3/TRAPPC2L/COL5A2/MDGA2/BRINP1/SPTSSB/KCNIP4/STAP1/TRPS1/UNC13B/ANO5/RBMS3/ITGA2B/CLRN3/DSG3/ODAPH/MARCH3/PRDM15/ISM1/LTK/SEMA5A/ATF6/SP1/CTC1/AAK1/SYT10/FAM47ESTBD1/FAM126A/RAB25/KCNN3/WBP1L/MED13/PRSS53/PAX7/PRRG4/PABPN1L/WNT9A/IGSF11/FDFT1/HTR2A/GFPT2/OCA2/CHL1/LMNB1*	GWAS Catalog[2]; Nat Genet. 2017 Oct;49(10):1511-1516[5].
Corticobasal degeneration	*MAPT /MOBP/SOS1/LINC02210-CRHR1/SPPL2C/KIF13B/DUSP4/TSPEAR*	GWAS Catalog[2]; Nat Commun. 2015 Jun 16;6:7247[6].
Progressive supranuclear palsy	*CD8B/IRF4/IL2/IL21/STX6/EIF2AK3/MOBP/MAPT/BMS1/SLCO1A2/EXOC2/KANSL1/NSF/WNT3/TRIM11/RUNX2/SP1/ASAP1/PIK3C2G*	GWAS Catalog[2]; Nat Genet. 2011 Jun 19;43(7):699-705[7].
Multiple systems atrophy	*FBXO47/ELOVL7/EDN1/CRHR1/MAPT/RREB1/ASB1/WNT3/MDGA2/CDH4/LOXL4/EBF2/CARD6/USP31/LMX1B/FBN2/XDH/SLC28A3/FOXL1/MPP6/ANKFN1/FOXN3/ENY2/NDE1/ARID1B/ASCC3/RIMS2/TENM2/DDX18/ASXL3/RASGRP3/ARHGAP44/ACOT11/ARL17A/RPL19/PLEKHM1/KANSL1/LINC02210CRHR1/PKHD1L1/PYROXD2/HS3ST2/THSD7B*	GWAS Catalog[2]; Neurology. 2016 Oct 11; 87(15): 1591–1598[8].
Frontotemporal dementia	*RAB38/CTSC/HLADRA/BTNL2/TMEM106B/VWDE/GFRA2/CEP131/WASHC5/NDUFAF8/TEPSIN/COL28A1/ST18/ATP9B/TNIK/RRBP1/BANF2/NDUFS1/C8orf49/NEIL2/FUT10/CFAP54/SEM1/DLD/HNF1B/CHL1/ZCCHC24/ACTN3/PALM2/SFRP4/STARD3NL/DMRT2/SLC38A10/UNC13A/TRMT11/ZFHX3/NECTIN2/LRRC37B/PRPF40A/SHANK3/SP1/FAT4*	GWAS Catalog[2]; Lancet Neurol. 2014 Jul;13(7):686-99[9].
Amyotrophic lateral sclerosis	*RPSA/SLC25A38/C9orf72/MOBP/SCFD1/POLDIP2/TMEM199/SARM1/KCNN1/KIF5A/MOB3B/UNC13A/ADGRD1/SUSD2/IDE/TBK1/C5orf30/OPCML/KIFAP3/TIAM1/ITGA9/CTNND2/ZNF746/TNIP1/ALCAM/CAMTA1/DPP6/WASHC5/ETNPPL/OLFM4/METTL21A/MASP1/CENPV/ATXN3/ANK3/PSD3/CPNE4/NPEPPS/KIAA0513/ARAP2/ABCG1/STK36/ZFYVE26/PBDC1/SLC9A9/NPS/LAMA3/CLVS1/KCNMB2/INPP4B/EFEMP1/NME9/ARHGEF2/SLC25A12/ERBB4/HOXD10/KALRN/KCNS3/IFRD1/SYNPO2/TBXAS1/ADAMTSL1/TMEM132B/NOG/CREB5/ITPR2/FOLH1B/TSPAN9/TFAP2A/STON1/PTH2R/HADH/LAMA2/GRID1/NEDD4L/MRAS/TBC1D1/ATXN1/SELL/TRPM8/CALN1/AS*	GWAS Catalog[2]; Nat Genet. 2016 Sep;48(9):1043-8[10]; Neuron. 2018 Mar 21;97(6):1268-1283.e6[11].

	IC2/PROCR/ZFP64/SQLE/PTPRF/CNOT2/C3orf56/ANXA3/RGS6/NFATC2/SUSD1/NRXN3/EPB41/CCSER1/PDLIM5/TAF8/PDGFRL/RBM19/CHODL/CNTN4/SCN7A/CTDSP1/PCSK5/ATP2B2/GSE1/DISC1/NT5C1A/SLC39A11/MORN2/COMMD10/CCDC192/ANKS1B/NFASC/MACROD2/ANKRD29/FHDC1/MIR99AHG/LDHC/DACH1/ABCC12/PPP2R2D/CFAP410/ABCC12/ST3GAL3/OSTC/PIGL/MAP3K7/DOCK1/MYOM2/PNPT1/ZBTB40/C17orf67/SEC16B/EGR1/SLC18A1/CALML3/STON1GTF2A1L/LEF1/WAPL/C1orf112/SPP2/KDM4A/LRRC8C/PLXNA1/XIRP2/VIL1/LIPC/RBMS1/ALDH1A2	
Huntington's disease	*FAN1/SOSTDC1/ISPD/ADGB/PTPRM/OR10A2/KIF9/BRF1/ADD1/TENM2/PTDSS1/EFR3A/MSH3/TRPM1/RNASET2/PRDM9/MTMR10/ATRNL1/GFRA1/ST8SIA6*	GWAS Catalog[2]; Lancet Neurol. 2017 Sep;16(9):701-711[12].
Alzheimer's disease	*PPOX/B4GALT3/ADAMTS4/NDUFS2/FCER1G/APOA2/CR1/CR1L/BIN1/ERCC3/GPR17/INPP5D/HESX1/MICB/C4A/C4B/PRRT1/EGFL8/AGPAT1/RNF5/AGER/PBX2/GPSM3/NOTCH4/TSBP1/BTNL2/HLA-DRA/HLA-DRB5/HLA-DRB1/HLA-DQA1/HLA-DQB1/HLA-DQA2/HLA-DQB2/HLA-DOB/TAP2/PSMB8/PSMB9/VPS52/TREML1/TREM2/CD2AP/ADGRF2/TRIM4/AZGP1/ZKSCAN1/ZSCAN21/COPS6/MCM7/AP4M1/TAF6/CNPY4/MBLAC1/LAMTOR4/C7orf43/GAL3ST4/GPC2/STAG3/CASTOR3/PVRIG/PILRB/PILRA/ZCWPW1/MEPCE/PPP1R35/C7orf61/TSC22D4/NYAP1/AGFG2/LRCH4/SAP25/FBXO24/MOSPD3/TFR2/GIGYF1/EPHB4/SLC12A9/FAM131B/ZYX/EPHA1/TAS2R60/ARHGEF35/OR2A7/CNTNAP2/TRIM35/PTK2B/CHRNA2/EPHX2/CLU/SCARA3/CCDC25/TCN1/MS4A3/MS4A2/MS4A6A/MS4A4E/MS4A4A/MS4A6E/PICALM/HIKESHI/SORL1/SLC24A4/RIN3/ADAM10/MINDY2/RNF111/SLTM/TPM1/LACTB/RAB8B/APH1B/CCDC189/RNF40/HSD3B7/STX1B/STX4/ZNF668/ZNF646/PRSS53/BCKDK/KAT8/PRSS8/PRSS36/ITGAX/MINK1/CHRNE/C17orf107/RNF167/CAMTA2/INCA1/KIF1C/ZFP3/ZNF232/USP6/SCIMP/RABEP1/NUP88/GNGT2/PHOSPHO1/ZNF652/ALPK2/FGF22/WDR18/GRIN3B/CNN2/ABCA7/ARHGAP45/POLR2E/GPX4/ZNF223/ZNF225/IGSF23/PVR/CEACAM19/CEACAM16/BCL3/CBLC/BCAM/NECTIN2/TOMM40/APOE/APOC1/APOC4/APOC2/CLPTM1/RELB/CLASRP/ZNF296/GEMIN7/MARK4/PPP1R37/NKPD1/TRAPPC6A/BLOC1S3/EXOC3L2/CKM/KLC3/ERCC2/EML2/FBXO46/DMPK/DMWD/IRF2BP1/CD33/SIGLECL1/VSIG10L/CSTF1/CASS4/RTF2/FAM209B/NECTIN2/APOC1P1/IL6R/MMP3/MMP12/ACKR2/KRBOX1/CYP8B1/UNC5CL/CCRL2/OARD1/CEACAM20/HNF4G/SUCLG2/BCAS3/BZW2/SPON1/FRMD4A/MTHFD1L/CR1/RBFOX1/ABI3/PLCG2/CLU/ATP5F1C/TMC5/CACNA1G/SYNJ1/GLIS3/CST1/GMNC/GPR141/ARL17B/PFDN1/NKAIN3/SDR42E2/FERMT2/PHF14/CTNNA2/ANKRD55/SAP30L/CELF1/NFIC/ITSN2/KDM1B/TMEM132C/MYO16/CLMN/MRPL58/IQCK/TGM6/ZNF292/CYYR1/EDAR/KAT8/PTPRG/LARS/TNRC6A/RAPSN/DSG2/BHMG1/OSBPL6/F13A1/FBXL7/CRADD/ACE/CEACAM19/ADGRF2/CASTOR3/SPI1/ZNF232/SNX9/COL12A1/CCDC89/FMN2/GPC6/VSTM2A/TGFB2/PNPLA7/SP6/NDUFAF6/LAMP1/FOXN2/AHNAK/TXNL1/GSK3B/DSCAML1/IL34/F2RL1/IQUB/CTHRC1/WAC/FAM181A/HERC2/TRPM1/MOBP/BMPER/TECTA/RNF165/IRF2/AKAP9/CELF2/CDC42SE2/ARVCF/SLC39A8/SETD7/FGF10/G3BP1/DST/EYA4/SGK3/PPP1R42/TRMO/SORCS3/CDH13/AFF1/CYCS/PCDH11X/MRPL39/ACTL8/COLGALT2/NIT2/CSMD1/MED12L/MEIKIN/UBXN11/TULP4/MICAL2/THSD4/ABCA8/SLC4A8/GABRG3/TLN2/IGHV270/SPPL2A/ARAP2/TRIQK/FNIP1/PLCL1/COL4A4/C3orf67/FAT1/TMED9/CDKAL1/AGBL1/ANO4/VAT1L/NDUFA12/TRIP4/GOLIM4/ANO3/RSPO4/KAZN/DYSF/FAM240B/C2orf76/ZCCHC10/AICDA/TEX33/DCHS2/STK32B/EXOC4/ST18/PARVB/ST6GAL1/OTOF/NR3C2/SH3RF1/SGK1/MPP3/LAMA1/ASIP/NKAIN2/PLPP4/VSNL1/AP2A2/C11orf65/IL19/SZT2/WDR41/CHN2/INSIG1/DCAF7/SIK1/NCS1/ARHGAP20/SQSTM1/LUZP2/SLC25A48/SESTD1/KRAS/COL25A1/HSPG2/MSH2/RAB1A/DLX5/PDCD1LG2/PPP4R3A/L3MBTL4/ZNF813/COL18A1/MEGF10/MECOM/KCNN2/FARP1/GGACT/PRRC2C/DMXL1/MAPK7/MPZL1/ATXN7L1/NCKAP5/ABCB11/EPC2/CCDC134/BLOC1S4/SH2D4B/CDH1/CEP295NL/PIFO/HMCN1/AHCYL1/SPRED2/C10orf71/DACH1/ELMO1/HECW1/ZAP70/FBXL13/EPOP/TNXB/LMOD3/SLC4A1AP/CCDC85C/SDR9C7/SLC14A2/SYPL1/FAM163A/NRXN1/HDAC9/KCNN3/PDE1A/SORD/RPS20P25/TMEM94/PCSK6/SLMAP/CSNK2A1/PTPRS/SCAPER/UGT1A10/CACNA2D3/LIMS2/CAMK4/PLEKHG1/RELN/FANCD2/RAB20/PDS5B/SPSB1/CDC42EP3/ADCY8/SLC44A5/SLC9A9/TNRC6C/RHBDF1/ELL/CENPM/SFT2D2/KBTBD8/CDON/RBMS3/LRAT/NARS2/BMP1/FBXO40/CASP12/TENM4/FAM19A5/RDX*	GWAS Catalog[2]; Nat Genet. 2019 Mar;51(3):404-413[13].

	/NAALADL2/JPH3/TBXAS1/HRK/BDH1/ADARB2/GRIN2B/EGLN3/ANTXR1/C9orf92/STK24/DVL2/ERBB4/DAPL1/ANKRD22/AKR7A3/CEP63/DLC1/RASSF8/RAPGEF5/SEC24B/TUSC1/PUM3/TOP1/MYRIP/PUS1/TMEM106B/SENP7/TRIM56/MAP4K4/RNF6/MGME1/LINC02210CRHR1/IRAK1BP1/BICRA/CCDC112/SELENOO/MDGA2/TFEB/DNAH6/LIN28B/C2orf83/PAK2/PCDH7/C5orf64/SLC2A9/PCNX1/POLN/WNT3/OFCC1/GPR180/MRPL45/CFAP74/SELP/USP10/RASSF5/KSR2/PAX2/ABCA1/MAP2K5/ANGPT4/ARIH1/CKAP5/ETS1/PEX6/TMCO4/TAS2R5/NEK10/MSX2/NEGR1/PKNOX2/CPM/CTNND2/AOX1/IQGAP2/PPP5D1/SYNGAP1/ERO1A/GTF2H3/SHANK2/C16orf95/TIAM2/NME9/NRXN3/FAM83E/RIMBP2/STK11/DIP2C/C5orf67/BMP2/CADM2/ADGRL2/SEMA3A/TXN/FAT3/KIFC3/SLC28A1/RBM19/DISC1/EFR3A/UTS2B/MPP7/IRF6/BSG/GAB2/GABRA2/TENT5A/OSTN/EPDR1/HBEGF/IL17RD/PDE7B/ADAMTS1/JCAD/PSMC3/NCR2/SLC10A2/ETF1/IL6/PMAIP1/F2R/CDC5L/FZD6/ACSL6/BANK1/ZNF862/FOXE1/SOBP/HTR7/PPP1R3B/EXOC1L/CDH19/CLDN18/STEAP3/CD24/DDX25/OTOGL/SEPT5/TST/RAPGEF6/ZNF117/CD300LG/TREML2/ATM/HYI/KCNK1/CADPS2/TSPAN16/CDR2L/KIAA0232/TIMP2/STRADA/YAP1/SRCIN1/FRMD4B/HMGA2/LINGO1/ZNF90/TCF15/SEM1/ZNRF4/UGT1A8/LIPC/FANCD2OS/ALDH4A1/MACROD2/RORA/PAX5/CCDC171/ZNF438/THSD7A/NHLRC3/SERPINE1/DIRAS2/OVOL2/BAALC/GDAP1L1/ZNF320/RPL3P8/KLHL36/MLN/LRIG3/PSMC6/LRRIQ3/SERINC5/CCZ1B/C9orf152/CNGB1/SPATA48/RAB3D/ALDH1A2/ZNF468/STEAP1B	
Dementia with Lewy body	*APOE/SNCA/KRTCAP2/BCL7C/FRMD3/NTS/RASSF9/HPS5/SAA1/MYO7A/HMX3/ACADSB/POT1/C7orf77/PARVB/PDLIM5/CTNND2/VPS36/GARNL3*	GWAS Catalog[2]

Supplementary Table 3. Disease associations of the human orthologs of aged mouse brain EC DEGs.

Human DEG orthologs	Disease / trait association	GWAS *P*-value	GWAS catalog accession ID / study
NBEAL1	White matter hyperintensity burden<br>Small-vessel ischemic stroke	5×10^{-8}<br>4×10^{-7}	GCST003013<br>GCST005841
SYNE2	Ischemic stroke	4.71×10^{-6}	Lancet Neurol. 2016 Jun; 15(7): 695–707[4].
VWF	von Willebrand factor levels in ischaemic stroke and hyperhomocysteinemia	5×10^{-6}	GCST004598
CDKN1A	Ischemic stroke	2×10^{-7}	GCST005843
SWAP70	Ischemic stroke	2×10^{-7}	GCST005843
SLC50A1	Parkinson's disease	5×10^{-8}	GCST001430
ALAS1	Parkinson's disease	3.2×10^{-8}	Nat Genet. 2017 Oct; 49(10): 1511–1516[5].
KTN1	Parkinson's disease	2×10^{-7}	GCST004902
TPM1	Parkinson's disease (age of onset)<br>Alzheimer's disease	9×10^{-11}<br>3.35×10^{-8}	GCST003652<br>Nat Genet. 2019 Mar;51(3):404-413[13].
PAM	Parkinson's disease	2×10^{-7}	GCST004902
ADAMTS1	Alzheimer's disease	3×10^{-8}	GCST007511
DLC1	Alzheimer's disease	4×10^{-6}	GCST003815
AHNAK	Age of onset in Alzheimer's disease<br>Alzheimer's disease	9×10^{-8}<br>2×10^{-7}	GCST003427<br>GCST005549
RDX	Alzheimer's disease	3×10^{-6}	GCST003815
IRF2	Hippocampal volume in Alzheimer's disease	2×10^{-7}	GCST006993
CD2AP	Alzheimer's disease	5×10^{-11}	GCST002245
GOLIM4	Cerebrospinal fluid total tau levels in Alzheimer's disease	5×10^{-7}	GCST006991
HMCN1	Alzheimer's disease	1×10^{-6}	GCST003815
MECOM	Alzheimer's disease	9×10^{-7}	GCST003815
SIK1	Age of onset in Alzheimer's disease	7×10^{-7}	GCST003427
PARVB	Alzheimer's disease	5×10^{-7}	GCST001915
TGFB2	Age of onset in Alzheimer's disease	8×10^{-8}	GCST003427
LIMS2	Alzheimer's disease	2×10^{-6}	GCST001915
CD42EP3	Total ventricular volume in Alzheimer's disease	2×10^{-6}	GCST000892
PRRC2C	Alzheimer's disease	9×10^{-7}	GCST001915
RORA	Total ventricular volume in Alzheimer's disease	3×10^{-6}	GCST000892
ADGRL2	Alzheimer's disease	9×10^{-6}	GCST003815
PAK2	Total ventricular volume in Alzheimer's disease	5×10^{-6}	GCST000892
KIF1C	Alzheimer's disease	9.16×10^{-10}	Nat Genet. 2019 Mar;51(3):404-413[13].
MPZL1	Alzheimer's disease	1×10^{-6}	GCST001658
RAPGEF5	Alzheimer's disease	4×10^{-6}	GCST003815

LEF1	Amyotrophic lateral sclerosis	3×10^{-6}	GCST002337
WAPL	Amyotrophic lateral sclerosis	3×10^{-6}	GCST002337
ATXN1	Amyotrophic lateral sclerosis	4×10^{-6}	GCST000406
ARHGEF2	Age of onset in amyotrophic lateral sclerosis	1×10^{-6}	GCST001663
EGR1	Amyotrophic lateral sclerosis	2×10^{-6}	GCST004791
SEM1	Age on onset in frontotemporal dementia<br>Alzheimer's disease	2×10^{-6}<br>2×10^{-6}	GCST006149<br>GCST003815
RRBP1	Frontotemporal dementia	1×10^{-6}	GCST006147
XDH	Multiple systems atrophy	6×10^{-6}	GCST003784
ASCC3	Multiple systems atrophy	3.66×10^{-6}	Neurology. 2016 Oct 11; 87(15): 1591–1598[8].

Supplementary Table 4. Evidence of human brain endothelial expression of the orthologs of a subset of aged mouse brain EC DEGs.

Human DEG Orthologs[i]	Concordant with normal aged brain	Concordant with AD brain	Confirmation of expression in human brain endothelium	Zone(s) of protein expression in endothelium[ii]	Other sites of protein expression[iii]
ABCB1	✓		Human Protein Atlas database[iv] showed expression in endothelium by IHC, also confirmed by another study[14].	A, C	None
ACER2	✓		Human Protein Atlas database showed expression in human cerebral cortex by bulk RT-PCR but IHC data was not available.<br>Allen Human Brain Cell Types snRNA-seq data[v] showed expression in endothelial cell with low counts.	Not applicable	Not applicable
ADAMTS1	✓		Human Protein Atlas database showed expression in human cerebral cortex by bulk RT-PCR, but IHC showed absent staining in endothelium.<br>Allen Human Brain Cell Types snRNA-seq data found no expression in endothelial cells.<br>Differentially upregulated in human glioma[15] and sporadic AD[16] but unclear if expressed in endothelium.	Not applicable	Not applicable
AHNAK	✓		Human Protein Atlas database showed expression in endothelium by IHC.	C, V	Neuronal cell bodies
APOLD1	✓		Human Protein Atlas database showed no expression in endothelial cells by IHC.<br>Allen Human Brain Cell Types snRNA-seq data showed expression in endothelial cell with high counts.	Not applicable	Neuronal cell bodies and nuclei
ARHGAP29		✓	Human Protein Atlas database showed expression in endothelium by IHC.	A, C, V	Neuronal cell bodies and neuropils
ARHGAP31	✓		Human Protein Atlas database showed expression in endothelium by IHC.	C	Neuronal cell bodies and neuropils, glial cells
ARL2BP	✓		Human Protein Atlas database showed expression in endothelium by IHC.	C	Neuronal cell bodies and neuropils
ATOX1		✓	Human Protein Atlas database showed expression in human cerebral cortex by bulk RT-PCR but IHC data was not available.<br>Allen Human Brain Cell Types snRNA-seq data did not find expression in endothelial cells.<br>No staining visualized by IHC even though clearly expressed in cerebral cortex by western blot[17], localized predominantly to neurons but also some endothelium in a study using in situ hybridization and IHC in rat brain[18].	Not applicable	Not applicable
CDKN1A	✕		Human Protein Atlas showed no expression in endothelium by IHC.<br>Allen Human Brain Cell Types snRNA-seq data showed expression in endothelial cells with low counts.<br>Expression might be inducible under certain conditions, predominantly in mitotic and/or tip cells of endothelium in mouse shown[19].	Not applicable	Neuronal cell bodies (only in certain samples)
CLDN5	✕		Human Protein Atlas database showed expression in endothelium by IHC, also shown in another study[20].	A, C, V	None
CXCL12	✕		Human Protein Atlas database showed expression in endothelium by IHC, confirmed by another study[21].	(very weak staining)	Neuronal cell bodies and some neuropils

DLC1		✓	Human Protein Atlas database showed expression in endothelium by IHC.	C	Certain glial cells
EPAS1	✓	✕	Human Protein Atlas database showed expression in human cerebral cortex by bulk RT-PCR but IHC data was not available.<br>Allen Human Brain Cell Types snRNA-seq data showed expression in endothelial cells with high counts, confirmed by another snRNA-seq study[22].	Not applicable	Not applicable
ESAM	✕		Human Protein Atlas database showed expression in endothelium by IHC.	A, C, V	Neuropils[vi]
FAS	✕		Human Protein Atlas database showed no expression in endothelial cells by IHC.<br>Allen Human Brain Cell Types snRNA-seq data showed no expression in endothelial cell.<br>Expressed in endothelial cells freshly isolated from human neurosurgical specimens performed for temporal lobe epilepsy[23].	Not applicable	None
FLT1	✓		Human Protein Atlas database showed expression in endothelium by IHC, confirmed in another study[24].	C, V	None[vii]
FOXQ1	✕		Human Protein Atlas database showed expression in human cerebral cortex by bulk RT-PCR but IHC data was not available.<br>Allen Human Brain Cell Types snRNA-seq data showed expression in endothelial cells with low counts.	Not applicable	Not applicable
GPR116	✓		Human Protein Atlas database showed expression in human cerebral cortex by bulk RT-PCR but IHC data was not available.<br>Expression data not available from Allen Human Brain Cell Types snRNA-seq database.<br>Expressed in mouse brain endothelial cells by RT-PCR[25] and in situ hybridization[26].	Not applicable	Not applicable
HES1	✓		Human Protein Atlas database showed expression in human cerebral cortex by bulk RT-PCR but IHC data was not available.<br>Allen Human Brain Cell Types snRNA-seq data showed expression in endothelial cells with low counts, confirmed by IF[27].	Uncertain, only endothelial cells in AVMs shown to express HES1	None[vii]
ID3	✕		Human Protein Atlas database showed expression in endothelium by IHC.	C[viii]	Neuronal cell bodies and neuropils, glial cells
IFITM3	✕	✓	Human Protein Atlas database showed expression in endothelium by IHC.	C, V	Neuronal cell bodies[ix]
IGF1R		✓	Human Protein Atlas database showed expression in endothelium by IHC in some samples.	(very weak staining)[x]	Neuronal cell bodies and neuropils
IGFBP7	✓		Human Protein Atlas database showed expression in endothelium by IHC, confirmed by another study[28].	(very weak staining)	Neuronal cell bodies
IQGAP1	✓	✓	Human Protein Atlas database showed expression in endothelium by IHC.	A, C, V	None
ITGA6	✓		Human Protein Atlas database showed expression in endothelium by IHC.	(very weak staining)[x]	Neuropils
KANK3	✕		Human Protein Atlas database showed expression in endothelium by IHC.	C, V	None
LIMA1		✕	Human Protein Atlas database showed expression in endothelium by IHC.	A, C	Neuronal cell bodies and neuropils, glial cells

LMO2	✓		Human Protein Atlas database showed expression in endothelium by IHC, confirmed by another study[29].	A, C, V	Neuronal cell bodies and neuropils, glial cells
LY6E	✓		Human Protein Atlas database showed expression in human cerebral cortex by bulk RT-PCR but IHC data was not available.<br>Allen Human Brain Cell Types snRNA-seq data showed expression in endothelial cells with low counts.	Not applicable	Not applicable
MECOM	✓		Human Protein Atlas database showed expression in endothelium by IHC, confirmed by IHC[30].	A, C, V	Neuronal cell bodies and neuropils, glial cells
MFSD2A		✓	Human Protein Atlas database showed expression in endothelium by IHC.	A, C	Neuronal cell bodies, glial cells, segmental neuropils
MYL12A	✕		Human Protein Atlas database showed expression in endothelium by IHC.	C	Neuropils
NOSTRIN	✕	✓	Human Protein Atlas database showed expression in endothelium by IHC.	C	Neuronal cell bodies and neuropils, glial cells
PLTP	✕		Annotated by authors of Human Protein Atlas to be absent in brain endothelial cells, but detailed inspection found there might be very faint staining in one of the images.<br>Allen Human Brain Cell Types snRNA-seq data showed no expression in endothelial cell. Also see another study for expression in human AD brains[31].	C	Neuronal cell bodies and neuropils
PTPRB	✓		Annotated by authors of Human Protein Atlas to be absent in brain endothelial cells, difficult to judge by inspection due to intense neuropil staining.<br>Allen Human Brain Cell Types snRNA-seq data showed expression in endothelial cells with low counts.	Not applicable	Neuropils
RGCC	✕		Human Protein Atlas database showed expression in endothelium by IHC.	A, C, V	Neuropils, neuronal cell bodies, glial cells
RHOC	✕		Human Protein Atlas database showed no expression in endothelium by IHC.<br>Allen Human Brain Cell Types snRNA-seq data showed expression in endothelial cell with low counts.	Not applicable	Not applicable
SDPR	✓		Human Protein Atlas database showed expression in endothelium by IHC.	C, V	None
SLC2A1	✕	✓	Human Protein Atlas database showed expression in endothelium by IHC, confirmed by another study[32].	C	Glial cells[xi]
SLC7A5	✕		Human Protein Atlas database showed expression in endothelium by IHC.	A, C	Neuronal cell bodies and neuropils
SLC9A3R2		✕	Human Protein Atlas database showed expression in endothelium by IHC, confirmed by another study[25].	A, C, V	None[vii]
TAGLN2	✕		Human Protein Atlas database showed expression in endothelium by IHC.	C	None[vii]
TIMP3	✓		Human Protein Atlas database showed expression in human cerebral cortex by bulk RT-PCR but IHC data was not available.	Not applicable	Not applicable

			Allen Human Brain Cell Types snRNA-seq data showed expression in endothelial cell with high counts.		
TINAGL1	×		Human Protein Atlas database showed expression in endothelium by IHC. Allen Human Brain Cell Types snRNA-seq data showed no expression in endothelial cells.	C	None
TSC22D1	×		Human Protein Atlas database showed expression in human cerebral cortex by bulk RT-PCR but IHC data was not available. Allen Human Brain Cell Types snRNA-seq data showed expression in endothelial cells with intermediate counts.	Not applicable	Not applicable
UACA	✓		Annotated by authors of Human Protein Atlas to be absent in brain endothelial cells, but detailed inspection found there might be weak staining in some segments of vessel endothelia.	A, C, V[xii]	Neuronal cell bodies
VWF	✓		Human Protein Atlas database showed expression in endothelium by IHC, confirmed by another study[33].	V[xii]	None[vii]

Abbreviations and annotations: A: arteriole, AD: Alzheimer's disease; C: capillary, DEG: differentially expressed genes, IF: immunofluorescence; IHC: immunohistochemistry, RT-PCR: reverse transcription-polymerase chain reaction, scRNA-Seq: single cell RNA sequencing; snRNA-seq: single nucleus RNA sequencing; V: venule; genes in bold font: have evidence of human brain endothelial expression.

Footnotes

i Gene names were chosen in accordance with Human Protein Atlas. Bold: confirmed expression in endothelium. Italic: unable to find evidence for endothelial expression.

ii Annotated by inspecting available human brain images from Human Protein Atlas and literature, may not be accurate due to sampling error.

iii Annotated by inspecting available human brain images from Human Protein Atlas and literature, cross-compared with Lee et al., 2017[34], may not be accurate due to sampling error.

iv Retrieved from https://www.proteinatlas.org/humanproteome/tissue/brain on 10/8/2019.

v Retrieved from https://celltypes.brain-map.org/rnaseq/human on 10/8/2019, data was based on snRNA-seq of cells from the middle temporal gyrus.

vi Lee et al 2017[34] annotated it as specific for endothelium, we however noted neuropil staining in all samples in Human Protein Atlas.

vii Not commented by Lee et al 2017[34] to be specific for endothelium.

viii Difficult to determine due to weak nuclear staining, not present in all samples.

ix Lee et al 2017[34] annotated it as specific for endothelium, we however noted that neuronal cell bodies were clearly stained in one sample in Human Protein Atlas and which appeared unlikely due to lipofuscin staining.

x Difficult to determine due to intense neuropil staining.

xi Lee et al 2017[34] annotated it as specific for endothelium by IHC, however a transmission electron microscopy study by Cornford et al 2005[32] noted potential glial expression.

xii Only in some segments of vessels.

Supplementary Table 5. Primer list.

Gene	Primer direction	Primer sequence (5' → 3')	T_m (°C)	Product size (bp)
Gapdh	Forward	GGCGGAGATGATGACCCTTT	59	145
	Reverse	CATCTTCCAGGAGCGAGACC		
Flt1	Forward	GTGTCTATAGGTGCCGAGCC	59	186
	Reverse	CGGAAGAAGACCGCTTCAGT		
Klf6	Forward	ACGAAACGGGCTACTTCTCG	59	324
	Reverse	TGGTCGTGGGTGAAAGTTCC		
Lef1	Forward	TCACTGTCAGGCGACACTTC	59	494
	Reverse	GCTGTCATTCTGGGACCTGT		
Smad7	Forward	CCTCGGAAGTCAAGAGGCTG	58	162
	Reverse	AGCCTGCAGTTGGTTTGAGA		
Mfsd2a	Forward	ACCTGAAACACCCTCACTCC	58	122
	Reverse	GTTGGCAAAGTCGAGACTGA		
Slc2a1	Forward	CTTGCTTGTAGAGTGACGATC	58	212
	Reverse	CAGTGATCCGAGCACTGCTC		

Supplementary Table 6. RNA FISH probe list.

mRNA target	FISH probe sequence (5' → 3'; one probe each row)
Afdn	GACTCCATGAAACTCCAAATCCTCGAAAA AAAgTCTAATCCgTCCCTgCCTCTATATCTCCACTC
	CCGAAACTCTTGCATTCTCTTCTCCAAAA AAAgTCTAATCCgTCCCTgCCTCTATATCTCCACTC
	ATCGGTAGTAGACAGCAGGATTGTCAAAA AAAgTCTAATCCgTCCCTgCCTCTATATCTCCACTC
	CGGTATAACTTTGGCTTGTCTCTGGAAAA AAAgTCTAATCCgTCCCTgCCTCTATATCTCCACTC
	CACAAACTTAAACACATGCGAGGTGCAAAA AAAgTCTAATCCgTCCCTgCCTCTATATCTCCACTC
	CTTGGAAAGAACGTGGTCCTGGATGAAAA AAAgTCTAATCCgTCCCTgCCTCTATATCTCCACTC
	GAAGAAAAGGCAGCTGTAGGTCTGGAAAA AAAgTCTAATCCgTCCCTgCCTCTATATCTCCACTC
	GATAGGATAGGCTTCAGGTCTGGGTAAAA AAAgTCTAATCCgTCCCTgCCTCTATATCTCCACTC
	CTGCGACTTTGAAGCTGGAAAGGTAAAAA AAAgTCTAATCCgTCCCTgCCTCTATATCTCCACTC
	CTGTGTTTTGAGGTAGGATGCGTTTCAAAA AAAgTCTAATCCgTCCCTgCCTCTATATCTCCACTC
	CTGCAGATCCCGAGTAAGAGTTTGGAAAA AAAgTCTAATCCgTCCCTgCCTCTATATCTCCACTC
	ATGTCAGATTTTCAGGGGCTCCAGAAAAA AAAgTCTAATCCgTCCCTgCCTCTATATCTCCACTC
	GTCACTGTCTGCTAACCAAATGGAGAAAA AAAgTCTAATCCgTCCCTgCCTCTATATCTCCACTC
	TCCATCCAGCCACGTCATACAAATGAAAA AAAgTCTAATCCgTCCCTgCCTCTATATCTCCACTC
	GTATGATTATGACACCTGAAGGCTGCAAAA AAAgTCTAATCCgTCCCTgCCTCTATATCTCCACTC
Lef1	TTACAATAGCTGGATGAGGGATGCCTTTT CACATTTACAgACCTCAACCTACCTCCAACTCTCAC
	CCTTTCTCTGTTCGTGTTGAGGCTTTTTT CACATTTACAgACCTCAACCTACCTCCAACTCTCAC
	CGTGCTAGTTCATAGTATTTGGCCTGTTTT CACATTTACAgACCTCAACCTACCTCCAACTCTCAC
	TCCTGTAGCTTCTCTCTCTTCCTCTTTTT CACATTTACAgACCTCAACCTACCTCCAACTCTCAC
	GACATGTACGGGTCGCTGTTCATATTTTT CACATTTACAgACCTCAACCTACCTCCAACTCTCAC
Ptprb	CTAAAGTTACAGGGTGACGCGAAAGTATA gCATTCTTTCTTgAggAgggCAgCAAACgggAAgAg
	CACGAGACCAGGAAATTAGGAGAGAATATA gCATTCTTTCTTgAggAgggCAgCAAACgggAAgAg
	ACAATAGTGAGGTTGTAGAGGTGGCTATA gCATTCTTTCTTgAggAgggCAgCAAACgggAAgAg
	CAGATTTGGCGATGATGAGGAACTGATATA gCATTCTTTCTTgAggAgggCAgCAAACgggAAgAg
	GGGGATGGTTTTGGTTTGAATGAAGCTATA gCATTCTTTCTTgAggAgggCAgCAAACgggAAgAg
	CCCCTGTTCACTGGCTTCATATTGTTATA gCATTCTTTCTTgAggAgggCAgCAAACgggAAgAg
	TTTCATGTCGTTGAAGAGCAGCTGGTATA gCATTCTTTCTTgAggAgggCAgCAAACgggAAgAg
	TCAGGACGAGGATTTTGTAATGGCGTATA gCATTCTTTCTTgAggAgggCAgCAAACgggAAgAg
	GCTCATAGAAGTCAAAGTCCCCGGATATA gCATTCTTTCTTgAggAgggCAgCAAACgggAAgAg
	CGAATGACAAAAGACTCGGAGGACTTATA gCATTCTTTCTTgAggAgggCAgCAAACgggAAgAg
	CCTTCTGACTTTCTGCTGCTGTAGGTATA gCATTCTTTCTTgAggAgggCAgCAAACgggAAgAg
	CAAAGTCGGAGTCAGGAGGTATCCATATA gCATTCTTTCTTgAggAgggCAgCAAACgggAAgAg
	GGATGAGATCACCGTAGTAGAGGGGTATA gCATTCTTTCTTgAggAgggCAgCAAACgggAAgAg
	CCATAAATGTCCACGGAGTCCTTAGTATA gCATTCTTTCTTgAggAgggCAgCAAACgggAAgAg
	AGGACGTCTCTTACACACTGATGCATATA gCATTCTTTCTTgAggAgggCAgCAAACgggAAgAg
Mfsd2a	CGCTAGCACTTAGCAAACCAGGAACTATA gCATTCTTTCTTgAggAgggCAgCAAACgggAAgAg
	GGTAGATCTGCAGGAAGAATCCCAGTTATA gCATTCTTTCTTgAggAgggCAgCAAACgggAAgAg
	GAGGAAGTAAGCAATGATGGCCAGGTATA gCATTCTTTCTTgAggAgggCAgCAAACgggAAgAg
	GTACCAAAGGAAGCCGTGTGAACTTTATA gCATTCTTTCTTgAggAgggCAgCAAACgggAAgAg
	TTGAATCGCTGTGCCTATCACTGTGTATA gCATTCTTTCTTgAggAgggCAgCAAACgggAAgAg
	ATTCTGGTCCTGGAGACAAGGTGCCTATA gCATTCTTTCTTgAggAgggCAgCAAACgggAAgAg
	ATTGGCAACTTCTGAGACCACCACATATA gCATTCTTTCTTgAggAgggCAgCAAACgggAAgAg

	AATGAAGGCACAGAGGACGTAGATGTATA gCATTCTTTCTTgAggAgggCAgCAAACgggAAgAg<br>CTGAAAGAAGGGCATTGACTCAGCCTATA gCATTCTTTCTTgAggAgggCAgCAAACgggAAgAg<br>GGTGCAGAACAAGGCAAAGTTACCCTATA gCATTCTTTCTTgAggAgggCAgCAAACgggAAgAg<br>GGATGGTGAATGTGGCCGAGAGCATTATA gCATTCTTTCTTgAggAgggCAgCAAACgggAAgAg<br>CAAACCGGGTTAGGAACCACTGCCATATA gCATTCTTTCTTgAggAgggCAgCAAACgggAAgAg<br>TGAGGGCCACCAAGATGAGAAAAGGTATA gCATTCTTTCTTgAggAgggCAgCAAACgggAAgAg<br>CATCGATAACGTCAGGCAGCATGGATATA gCATTCTTTCTTgAggAgggCAgCAAACgggAAgAg<br>CAGGGGAGTGAGGGTGTTTCAGGTGTATA gCATTCTTTCTTgAggAgggCAgCAAACgggAAgAg<br>CCAGAGGCAAACTTGGTGAAGAAGACTATA gCATTCTTTCTTgAggAgggCAgCAAACgggAAgAg<br>CATCCCTGCCTCTGGTAGTTGGCAATATA gCATTCTTTCTTgAggAgggCAgCAAACgggAAgAg<br>GATGATAGGAGCCATGGTCACCAGCTATA gCATTCTTTCTTgAggAgggCAgCAAACgggAAgAg<br>TTTCTTATTCTGTCGCCGCTTCTCCTATA gCATTCTTTCTTgAggAgggCAgCAAACgggAAgAg<br>TGTGGAGTCTGTATCCGAGCAACCTTATA gCATTCTTTCTTgAggAgggCAgCAAACgggAAgAg<br>AGGTCCTTTGTGTGGTGGCTTCAGGTATA gCATTCTTTCTTgAggAgggCAgCAAACgggAAgAg<br>GATCCCGTGCTTAATGCTACCTGGTTATA gCATTCTTTCTTgAggAgggCAgCAAACgggAAgAg
Smad7	CGTATCTGGGTGTTTTAGAGTCTCTGAAAA AAAgTCTAATCCgTCCCTgCCTCTATATCTCCACTC<br>CCGGTCTTCCTTTCCTTTTCTTCTGAAAA AAAgTCTAATCCgTCCCTgCCTCTATATCTCCACTC<br>CTTAGTAACCGGATCTCCTCGAATTCAAAA AAAgTCTAATCCgTCCCTgCCTCTATATCTCCACTC<br>GGTGGGGAGAAGCAGGCAATTAAAAAAAA AAAgTCTAATCCgTCCCTgCCTCTATATCTCCACTC<br>GGCTGAGCTACTAAAGGGAGGAAAAAAAA AAAgTCTAATCCgTCCCTgCCTCTATATCTCCACTC<br>ATTCACAGCAACACAGCCTCTTGACAAAA AAAgTCTAATCCgTCCCTgCCTCTATATCTCCACTC<br>CTGACTCTTGTTGTCCGAATTGAGCAAAA AAAgTCTAATCCgTCCCTgCCTCTATATCTCCACTC<br>GGACTTGATGAAGATGGGGTAACTGAAAA AAAgTCTAATCCgTCCCTgCCTCTATATCTCCACTC<br>TCGGGAAAGGTTAGCAGCAAGTAGTAAAA AAAgTCTAATCCgTCCCTgCCTCTATATCTCCACTC<br>TCGTTTCCTGTCTTCGTATATGGGGAAAA AAAgTCTAATCCgTCCCTgCCTCTATATCTCCACTC<br>TAGAGAGACACACAGCTTGGCACTAAAAA AAAgTCTAATCCgTCCCTgCCTCTATATCTCCACTC<br>GCGGCAGTAAGACAGGGATGAAAACAAAA AAAgTCTAATCCgTCCCTgCCTCTATATCTCCACTC<br>AAGAGAGAGATCCAGGAGCAGATGGAAAA AAAgTCTAATCCgTCCCTgCCTCTATATCTCCACTC<br>GTACTGGGGTAGCAATACTGTGACTAAAA AAAgTCTAATCCgTCCCTgCCTCTATATCTCCACTC<br>GTCCTTTCCTCTCTCAAAGCACTACAAAA AAAgTCTAATCCgTCCCTgCCTCTATATCTCCACTC
Flt1	CAGAATTGCCTGTTATCCCTCCCACTATA gCATTCTTTCTTgAggAgggCAgCAAACgggAAgAg<br>CAAGTTTGGGTATGTCAGTGTGCATCTATA gCATTCTTTCTTgAggAgggCAgCAAACgggAAgAg<br>TGATGAGCTGTCTTCCTTCCGTCATTATA gCATTCTTTCTTgAggAgggCAgCAAACgggAAgAg<br>AAGCCTCTCCTACTGTCCCATGTTATATA gCATTCTTTCTTgAggAgggCAgCAAACgggAAgAg<br>GTTCAGCAGTCCTATCTCTTTGTACGTATA gCATTCTTTCTTgAggAgggCAgCAAACgggAAgAg<br>TCATTTGCACCCTCGTATTGAGCTCTATA gCATTCTTTCTTgAggAgggCAgCAAACgggAAgAg<br>TCTCGGCTCTCCACATTGTTGATCTTATA gCATTCTTTCTTgAggAgggCAgCAAACgggAAgAg<br>GAGGTGTTGAAAGACTGGAACGAGGTATA gCATTCTTTCTTgAggAgggCAgCAAACgggAAgAg<br>CTTCACTTTCATGGACAGCCGATAGTATA gCATTCTTTCTTgAggAgggCAgCAAACgggAAgAg<br>CATGTACCAAATAGCGAGCAGACTTCTATA gCATTCTTTCTTgAggAgggCAgCAAACgggAAgAg<br>CTGCATCCTCGGTTGTCACATCTTTTATA gCATTCTTTCTTgAggAgggCAgCAAACgggAAgAg<br>CCTTTCTTTGGAGTGATTGTGGTGACAGTATA gCATTCTTTCTTgAggAgggCAgCAAACgggAAgAg<br>AGTCCCTATTTTATTGAAGGCCCGGTATA gCATTCTTTCTTgAggAgggCAgCAAACgggAAgAg<br>TCCGTAGCAGAATCCAGGTAATGTCTATA gCATTCTTTCTTgAggAgggCAgCAAACgggAAgAg<br>TATATGTTCCTGGCTCTGCACGCATATATA gCATTCTTTCTTgAggAgggCAgCAAACgggAAgAg

Ifitm3	GTGATGAAGGCTTGAGAAGTGTGGTTAAAA AgCTCAgTCCATCCTCgTAAATCCTCATCAATCATC<br>CTCAGCCACCTCATATTCTTCCTTGAAAAA AgCTCAgTCCATCCTCgTAAATCCTCATCAATCATC
Jund	CAGAGGAGAGTTGGGAGTCTGTTCTAAAA AgCTCAgTCCATCCTCgTAAATCCTCATCAATCATC<br>CTCTTAACCTCGGCCTACAGATCTCAAAA AgCTCAgTCCATCCTCgTAAATCCTCATCAATCATC<br>CCCTGTTTCATATTCCCAAGTGTGGAAAA AgCTCAgTCCATCCTCgTAAATCCTCATCAATCATC<br>CTCATCCTCTAGACTCCGCTATTCATAAAA AgCTCAgTCCATCCTCgTAAATCCTCATCAATCATC<br>CAGGGTAGCCATCAGTTGTAGGTTTAAAA AgCTCAgTCCATCCTCgTAAATCCTCATCAATCATC<br>AAACTGAGGATGGGATGTGTGGAGTAAAA AgCTCAgTCCATCCTCgTAAATCCTCATCAATCATC<br>TGGACCTCATCTGTGCGTCTATCTCAAAA AgCTCAgTCCATCCTCgTAAATCCTCATCAATCATC<br>CCTCTAGATCTTGCCTCTCTGTTTCTAAAA AgCTCAgTCCATCCTCgTAAATCCTCATCAATCATC<br>CTCCCTTTCTCTTCCTCCCCTCTTTAAAA AgCTCAgTCCATCCTCgTAAATCCTCATCAATCATC<br>GTGACGTCGTTGATTTGCATAAAGGGAAAA AgCTCAgTCCATCCTCgTAAATCCTCATCAATCATC<br>GCTTTTGAGGGTCTTGACTTTCTCCTAAAA AgCTCAgTCCATCCTCgTAAATCCTCATCAATCATC<br>GTGGCTGAGGACTTTCTGTTTGAGCAAAA AgCTCAgTCCATCCTCgTAAATCCTCATCAATCATC
Slc9a3r2	GTGTTCATCAGTCTCAGGATCGACTAAAAA AAAgTCTAATCCgTCCCTgCCTCTATATCTCCACTC<br>ATGTTCCTCAGTGGGTATGACCCTCAAAA AAAgTCTAATCCgTCCCTgCCTCTATATCTCCACTC<br>CTCTCTGATCTTGCTATGACCACCGAAAA AAAgTCTAATCCgTCCCTgCCTCTATATCTCCACTC<br>CTCTGTCTCTCTCTTCTCTGGGTTTAAAA AAAgTCTAATCCgTCCCTgCCTCTATATCTCCACTC<br>TCACTGTTTCTGTCTCCGTGTCTGTAAAA AAAgTCTAATCCgTCCCTgCCTCTATATCTCCACTC<br>ACTAGAAACATGTGGAGCTGGAGACAAAA AAAgTCTAATCCgTCCCTgCCTCTATATCTCCACTC<br>GGTGGGTAAGAAAGGGTTTGCAGAGAAAA AAAgTCTAATCCgTCCCTgCCTCTATATCTCCACTC<br>CTGAGTAAGCCCCAGTTGAGAGTTCAAAA AAAgTCTAATCCgTCCCTgCCTCTATATCTCCACTC<br>CTCCTCTCTGATCTTGCTATGACCAAAAA AAAgTCTAATCCgTCCCTgCCTCTATATCTCCACTC<br>TATCTCTGTCTCTCTCTTCTCTGGGAAAA AAAgTCTAATCCgTCCCTgCCTCTATATCTCCACTC<br>CTGTTTCTGTCTCCGTGTCTGTCTTAAAA AAAgTCTAATCCgTCCCTgCCTCTATATCTCCACTC<br>ACTAGAAACATGTGGAGCTGGAGACAAAA AAAgTCTAATCCgTCCCTgCCTCTATATCTCCACTC
Nostrin	CTTTGGCGTAGCTAATCTCCAGGTTTATA gCATTCTTTCTTgAggAgggCAgCAAACgggAAgAg<br>TTGTGCTCTGTAGTGCTTTGCTGAGTATA gCATTCTTTCTTgAggAgggCAgCAAACgggAAgAg<br>CTCTTCTTCTCTTGCATACTCAGGACTATA gCATTCTTTCTTgAggAgggCAgCAAACgggAAgAg<br>AATTCTTTCCTTCTCCAGCTCCAGCTATA gCATTCTTTCTTgAggAgggCAgCAAACgggAAgAg<br>GGATTGCAGTTTCTTCCATTAGAGCCTATA gCATTCTTTCTTgAggAgggCAgCAAACgggAAgAg<br>CCATGTCTTTCTGGCTCTTGGCATCTATA gCATTCTTTCTTgAggAgggCAgCAAACgggAAgAg<br>AGGAGTTCGCTTGCAAAAGGTCTAGTATA gCATTCTTTCTTgAggAgggCAgCAAACgggAAgAg<br>ACTTGAAAATGCAGGTGCTACAGGGTATA gCATTCTTTCTTgAggAgggCAgCAAACgggAAgAg<br>GTCAAAAGAGGCCGGGATATTTTCACTATA gCATTCTTTCTTgAggAgggCAgCAAACgggAAgAg<br>GAAGAGCTTGGATTGTTCTGCCCACTATA gCATTCTTTCTTgAggAgggCAgCAAACgggAAgAg<br>GGCTTGGAACGTATATAAGGCTTTGCTATA gCATTCTTTCTTgAggAgggCAgCAAACgggAAgAg<br>TTCTTTCTTCTCATGGACGGTCACGTATA gCATTCTTTCTTgAggAgggCAgCAAACgggAAgAg<br>CCCTTTTAAAGAGCCGAACCACCATTATA gCATTCTTTCTTgAggAgggCAgCAAACgggAAgAg<br>CCTCTACCACCCACTGCATAAGAAAATATA gCATTCTTTCTTgAggAgggCAgCAAACgggAAgAg

Supplementary References

1. Vanlandewijck, M. *et al.* A molecular atlas of cell types and zonation in the brain vasculature. *Nature* **554**, 475 (2018).
2. Buniello, A. *et al.* The NHGRI-EBI GWAS Catalog of published genome-wide association studies, targeted arrays and summary statistics 2019. *Nucleic Acids Res* **47**, D1005–D1012 (2018).
3. Verhaaren, B. *et al.* Multiethnic Genome-Wide Association Study of Cerebral White Matter Hyperintensities on MRI. *Circulation: Cardiovascular Genetics* **8**, 398–409 (2015).
4. Consortium, N. of the for and in, (SiGN), S. & (ISGC), I. Identification of additional risk loci for stroke and small vessel disease: a meta-analysis of genome-wide association studies. *The Lancet Neurology* **15**, 695–707 (2016).
5. Chang, D. *et al.* A meta-analysis of genome-wide association studies identifies 17 new Parkinson's disease risk loci. *Nat Genet* **49**, 1511–1516 (2017).
6. Kouri, N. *et al.* Genome-wide association study of corticobasal degeneration identifies risk variants shared with progressive supranuclear palsy. *Nat Commun* **6**, 7247 (2015).
7. Höglinger, G. U. *et al.* Identification of common variants influencing risk of the tauopathy progressive supranuclear palsy. *Nat Genet* **43**, 699 (2011).
8. Sailer, A. *et al.* A genome-wide association study in multiple system atrophy. *Neurology* **87**, 1591–1598 (2016).
9. Ferrari, R. *et al.* Frontotemporal dementia and its subtypes: a genome-wide association study. *The Lancet. Neurology* **13**, 686–99 (2014).
10. Rheenen, W. van *et al.* Genome-wide association analyses identify new risk variants and the genetic architecture of amyotrophic lateral sclerosis. *Nature Genetics* **48**, 1043 (2016).
11. Nicolas, A. *et al.* Genome-wide Analyses Identify KIF5A as a Novel ALS Gene. *Neuron* **97**, 1268-1283.e6 (2018).
12. Moss, D. J. *et al.* Identification of genetic variants associated with Huntington's disease progression: a genome-wide association study. *Lancet Neurology* **16**, 701–711 (2017).
13. Jansen, I. E. *et al.* Genome-wide meta-analysis identifies new loci and functional pathways influencing Alzheimer's disease risk. *Nature Genetics* **51**, 404–413 (2019).
14. Bendayan, R., Ronaldson, P. T., Gingras, D. & Bendayan, M. In Situ Localization of P-glycoprotein (ABCB1) in Human and Rat Brain. *J Histochem Cytochem* **54**, 1159–1167 (2006).
15. Martino-Echarri, E. *et al.* Relevance of IGFBP2 proteolysis in glioma and contribution of the extracellular protease ADAMTS1. *Oncotarget* **5**, 4295–4304 (2014).
16. Medoro, A. *et al.* Proteases Upregulation in Sporadic Alzheimer's Disease Brain. *J Alzheimer's Dis* **68**, 931–938 (2019).
17. Davies, K. M. *et al.* Localization of copper and copper transporters in the human brain. *Metallomics* **5**, 43–51 (2013).
18. Naeve, G. S. *et al.* Expression profile of the copper homeostasis gene, rAtox1, in the rat brain. *Neuroscience* **93**, 1179–1187 (1999).
19. Sabbagh, M. F. *et al.* Transcriptional and epigenomic landscapes of CNS and non-CNS vascular endothelial cells. *Elife* **7**, e36187 (2018).
20. Du, Y. *et al.* Increased cerebral expressions of MMPs, CLDN5, OCLN, ZO1 and AQPs are associated with brain edema following fatal heat stroke. *Sci Rep-uk* **7**, 1691 (2017).
21. Krumbholz, M. *et al.* Chemokines in multiple sclerosis: CXCL12 and CXCL13 up-regulation is differentially linked to CNS immune cell recruitment. *Brain* **129**, 200–211 (2005).
22. Grubman, A. *et al.* A single-cell atlas of entorhinal cortex from individuals with Alzheimer's disease reveals cell-type-specific gene expression regulation. *Nat Neurosci* **22**, 2087–2097 (2019).
23. Wosik, K., Biernacki, K., Khouzam, M.-P. & Prat, A. Death receptor expression and function at the human blood brain barrier. *J Neurol Sci* **259**, 53–60 (2007).
24. Uranishi, R., Baev, N. I., Ng, P.-Y., Kim, J. H. & Awad, I. A. Expression of Endothelial Cell Angiogenesis Receptors in Human Cerebrovascular Malformations. *Neurosurgery* **48**, 359–368 (2001).

25. Wallgard, E. *et al.* Identification of a Core Set of 58 Gene Transcripts With Broad and Specific Expression in the Microvasculature. *Arteriosclerosis Thrombosis Vasc Biology* **28**, 1469–1476 (2008).
26. Ehrlich, A. T. *et al.* Expression map of 78 brain-expressed mouse orphan GPCRs provides a translational resource for neuropsychiatric research. *Commun Biology* **1**, 102 (2018).
27. Murphy, P. A., Lu, G., Shiah, S., Bollen, A. W. & Wang, R. A. Endothelial Notch signaling is upregulated in human brain arteriovenous malformations and a mouse model of the disease. *Laboratory Investigation J Technical Methods Pathology* **89**, 971–82 (2009).
28. Iqbal, U. *et al.* Molecular imaging of glioblastoma multiforme using anti-insulin-like growth factor-binding protein-7 single-domain antibodies. *Brit J Cancer* **103**, 1606–16 (2010).
29. Gratzinger, D. *et al.* The transcription factor LMO2 is a robust marker of vascular endothelium and vascular neoplasms and selected other entities. *Am J Clin Pathol* **131**, 264–78 (2009).
30. Hou, A. *et al.* Expression of MECOM is associated with unfavorable prognosis in glioblastoma multiforme. *Oncotargets Ther* **9**, 315–20 (2016).
31. Vuletic, S. *et al.* Widespread distribution of PLTP in human CNS: evidence for PLTP synthesis by glia and neurons, and increased levels in Alzheimer's disease. *J Lipid Res* **44**, 1113–1123 (2003).
32. Cornford, E. M. & Hyman, S. Localization of brain endothelial luminal and abluminal transporters with immunogold electron microscopy. *Neurorx* **2**, 27–43 (2005).
33. Macdonald, J. A., Murugesan, N. & Pachter, J. S. Endothelial cell heterogeneity of blood-brain barrier gene expression along the cerebral microvasculature. *J Neurosci Res* **88**, NA-NA (2009).
34. Lee, S. J. *et al.* Large-scale identification of human cerebrovascular proteins: Inter-tissue and intracerebral vascular protein diversity. *Plos One* **12**, e0188540 (2017).
