# Supplementary material for: Pharmacologically reversible zonation-dependent endothelial cell transcriptomic changes with neurodegenerative disease associations in the aged brain
Source: Nat Commun. 2020 Sep 4;11:4413. doi: 10.1038/s41467-020-18249-3 (PMC7474063; doi:10.1038/s41467-020-18249-3)
Supplement: Supplementary file 3 — Reporting Summary [file 41467_2020_18249_MOESM3_ESM.pdf]

nature research

Corresponding author(s): Ho Ko

Last updated by author(s): Aug 5, 2020

Reporting Summary

Nature Research wishes to improve the reproducibility of the work that we publish. This form provides structure for consistency and transparency in reporting. For further information on Nature Research policies, see Authors & Referees and the Editorial Policy Checklist .

Statistics

For all statistical analyses, confirm that the following items are present in the figure legend, table legend, main text, or Methods section.

n/a	Confirmed	
☐	☒	The exact sample size (n) for each experimental group/condition, given as a discrete number and unit of measurement
☐	☒	A statement on whether measurements were taken from distinct samples or whether the same sample was measured repeatedly
☐	☒	The statistical test(s) used AND whether they are one- or two-sided<br>*Only common tests should be described solely by name; describe more complex techniques in the Methods section.*
☐	☒	A description of all covariates tested
☐	☒	A description of any assumptions or corrections, such as tests of normality and adjustment for multiple comparisons
☐	☒	A full description of the statistical parameters including central tendency (e.g. means) or other basic estimates (e.g. regression coefficient) AND variation (e.g. standard deviation) or associated estimates of uncertainty (e.g. confidence intervals)
☐	☒	For null hypothesis testing, the test statistic (e.g. F, t, r) with confidence intervals, effect sizes, degrees of freedom and P value noted<br>*Give P values as exact values whenever suitable.*
☒	☐	For Bayesian analysis, information on the choice of priors and Markov chain Monte Carlo settings
☒	☐	For hierarchical and complex designs, identification of the appropriate level for tests and full reporting of outcomes
☐	☒	Estimates of effect sizes (e.g. Cohen's d, Pearson's r), indicating how they were calculated

Our web collection on statistics for biologists contains articles on many of the points above.

Software and code

Policy information about availability of computer code

Data collection	For mouse brain cell RNA sequencing, the raw fastq files were aligned by Cell Ranger (v3.0.0, 10X Genomics, US) and converted to count matrix for further processing.
Data analysis	The sequencing data processing were performed with the Seurat (v3.0.1) package and custom scripts in R (v3.5.1).<br>For differential expression analysis, the MAST (v1.8.2) package was used (which was already incorporated in the Seurat package).<br>Figures were generated by the Seurat package, the ggplot2 (3.1.1) package, and custom scripts in R (v3.5.1).<br>For two-photon imaging data processing, the Imaris software (v6.4, Bitplane, Belfast, UK) was used.<br>Immunohistochemistry data was analyzed using the Fiji (ImageJ) software with cell counter plugin (64-bit version based on ImageJ 1.52p).<br>Statistical tests were carried out in R (v3.5.1) or the Prism software (v6, GraphPad Software Inc., US).

For manuscripts utilizing custom algorithms or software that are central to the research but not yet described in published literature, software must be made available to editors/reviewers. We strongly encourage code deposition in a community repository (e.g. GitHub). See the Nature Research guidelines for submitting code & software for further information.

Data

Policy information about availability of data

All manuscripts must include a data availability statement. This statement should provide the following information, where applicable:

- Accession codes, unique identifiers, or web links for publicly available datasets
- A list of figures that have associated raw data
- A description of any restrictions on data availability

The raw and processed RNA sequencing data from this study have been deposited in the UCSC Cell Browser (“Aging Brain [http://cells.ucsc.edu/?ds=aging-brain]”), the Broad Institute Single Cell Portal (“Aging_mouse_brain_kolab [https://singlecell.broadinstitute.org/single_cell/study/SCP829/aging-mouse-brain-kolab]”) and the NCBI Gene Expression Omnibus database (accession number: GSE147693).
For human aged brain transcriptome comparative analysis, the GTEx datasets were downloaded from [https://www.gtexportal.org/home/datasets]

(GTEx_Analysis_2016-01-15_v7_RNASeQCv1.1.8_gene_tpm.gct.gz and GTEx_Analysis_2017-06-05_v8_RNASeQCv1.1.9_gene_tpm.gct.gz) alongside subject information.
For the Allen Brain Aging, Dementia and TBI dataset, the normalized FPKM data matrix was downloaded from [https://aging.brain-map.org/download/index] alongside subject information.
For the Mayo Clinic AD brain RNA-seq dataset and DEGs, data was downloaded from [http://dx.doi.org/10.7303/syn3163039] alongside subject information.
Source data (underlying Figs 2d-e, 4b-c, and Supplementary Figs 7b, 9b) are provided with this paper.

Field-specific reporting

Please select the one below that is the best fit for your research. If you are not sure, read the appropriate sections before making your selection.

☒ Life sciences ☐ Behavioural & social sciences ☐ Ecological, evolutionary & environmental sciences

For a reference copy of the document with all sections, see nature.com/documents/nr-reporting-summary-flat.pdf

Life sciences study design

All studies must disclose on these points even when the disclosure is negative.

Sample size

We used 5 mice per group (young adult, aged and exenatide-treated aged groups) for the the scRNA-seq experiments and 3 mice per group for the imaging experiments (young adult, aged, vehicle-treated and exenatide-treated aged groups, with two batches of experiments done). For the qPCR and WB experiments, 3 mice for each group (young adult and aged groups) were used for each experiment. For the IHC experiments, 4, 3 and 3 animals for the young adult, aged, and exenatide-treated aged groups were used respectively. No prior sample size calculation was performed. Selection of sample sizes was based on past research experience judging potential biological effects relative to expected variability, typically requiring 3-5 animals per group for the types of experiments carried out.

Data exclusions

For sequencing data quality control, genes expressed by fewer than 3 cells were excluded; low quality cells were excluded by the following criteria: 1. lower than 5% or higher than 95% UMI count or gene count, or 2. proportion of mitochondrial genes > 20%. This was a pre-established criteria.
For differential expression analysis, endothelial cells with unstable subtype classification on three independent repeated runs of CellAssign were excluded (1074 out of 12357, 8.7%). This was a pre-established criteria.
For WB, we assayed the expression changes of LEF1, SMAD7, MFSD2A and SLC2A1 across age. The antibodies we used worked for LEF1, SMAD7 and MFSD2A, but very broad and possibly non-specific binding bands were noted for the anti-SLC2A1 in our experiment (see Source Data file for uncropped WB images for Fig. 2e, rightmost image), hence only the results of LEF1, SMAD7 and MFSD2A were included in the main figure.

Replication

Repeating independent batches of experiments was the major means to ensure reproducibility (i.e. instead of relying on data from one single experiment). The sequencing data presented came from three independent batches of experiments with a total of 5 animal subjects used for each group. The in vivo imaging experiments were carried out in two independent batches. The first batch (with 3 animals per group) was performed to test the therapeutic efficacy of exenatide treatment. During each imaging session, 3 image stacks were taken from each animal within the specified time window (i.e. 15-35 mins post-IV dextran dye injection), thereby serving as replicates of sampling for each animal. In the second batch, additional experiments (also with 3 animals per group) were carried out with identical protocol to verify the therapeutic efficacy of exenatide over saline vehicle.
Analysis of data from the different batches of experiments arrived at the same conclusion on the therapeutic efficacy of exenatide treatment. The consistency of findings hence support the reproducibility of the findings. We confirm all attempts at replication were successful for all the experiments.

Randomization

Not required. This study involved comparison across age and treatment groups, and therefore samples were grouped by age and treatment as the primary distinguishing parameters.

Blinding

Not required. This study did not involve subjective measurements as all sequencing and imaging data were included with identical and strict criteria, and analyzed by identical software or programming pipelines.

Reporting for specific materials, systems and methods

We require information from authors about some types of materials, experimental systems and methods used in many studies. Here, indicate whether each material, system or method listed is relevant to your study. If you are not sure if a list item applies to your research, read the appropriate section before selecting a response.

Materials & experimental systems

n/a	Involved in the study
☐	☒ Antibodies
☒	☐ Eukaryotic cell lines
☒	☐ Palaeontology
☐	☒ Animals and other organisms
☒	☐ Human research participants
☒	☐ Clinical data

Methods

n/a	Involved in the study
☒	☐ ChIP-seq
☒	☐ Flow cytometry
☒	☐ MRI-based neuroimaging

Antibodies

Antibodies used

Anti-GAPDH: Abcam, Cat#ab8245, clone 6C5, Lot#GR291713-2.
Anti-LEF-1: Santa Cruz Biotechnology, Cat#sc-374522, clone B6, Lot# N/A.
Anti-SMAD-7: Santa Cruz Biotechnology, Cat#sc-101152, clone Z8-B, Lot#I0619.
Anti-MFSD2A: Thermo Fisher Scientific, Cat#PA521049, polyclonal, Lot#VA2925285.
Anti-IBA1: Wako Chemicals, Cat#019-19741, polyclonal, Lot#CAJ3125.
Anti-SLC2A1: Abcam, Cat#ab652, polyclonal, Lot#GR3274850.
Anti-CD45: BD Pharmingen, Cat#550539, clone 30-F11, Lot#8018762
Anti-CD31: BD Pharmingen, Cat#553370, clone MEC 13.3, Lot#9338730
Alexa Fluor 488-conjugated anti-rabbit: Thermo Fisher Scientific, Cat#A-21206, polyclonal, Lot#1834802

Validation

Anti-GAPDH: has been validated by manufacturer for WB application against mouse GAPDH protein.
Anti-LEF-1: has been validated by manufacturer for WB application against mouse LEF-1 protein.
Anti-SMAD-7: has been validated by manufacturer for WB application against mouse SMAD-7 protein.
Anti-MFSD2A: has been validated by manufacturer for WB application against mouse MFSD2A protein.
Anti-IBA1: has been validated by manufacturer for IHC application against mouse tissues.
Anti-SLC2A1: has been validated by manufacturer for WB application against mouse SLC2A1 protein.
Anti-CD45: has been validated by manufacturer for flow cytometry application against mouse cells and by previous report (doi: 10.1101/pdb.prot074963) for immunopanning of mouse microglia.
Anti-CD31: has been validated by manufacturer for flow cytometry application against mouse cells and by previous report (doi: 10.1101/pdb.prot074963) for immunopanning of mouse brain endothelial cells.
Alexa Fluor 488-conjugated anti-rabbit antibody: has been validated by manufacturer for immunofluorescence application.

Animals and other organisms

Policy information about studies involving animals; ARRIVE guidelines recommended for reporting animal research

Laboratory animals

Male C57BL/6J mice aged 2-3 months old and 18-20 months old, provided by the the Laboratory Animal Service Center of the Chinese University of Hong Kong and maintained at controlled temperature (22 – 23°C) with an alternating 12-hour light/dark cycle with free access to standard mouse diet and water. The ambient humidity was maintained at < 70 % relative humidity.

Wild animals

The study did not involve wild animals.

Field-collected samples

The study did not use samples collected from the field.

Ethics oversight

All experimental procedures were approved in advance by the Animal Research Ethical Committee of the Chinese University of Hong Kong (CUHK) and were carried out in accordance with the Guide for the Care and Use of Laboratory Animals.

Note that full information on the approval of the study protocol must also be provided in the manuscript.
